# Supplementary material for: Nanoparticles exhibiting virus-mimic surface topology for enhanced oral delivery
Source: Nat Commun. 2023 Nov 24;14:7694. doi: 10.1038/s41467-023-43465-y (PMC10673925; doi:10.1038/s41467-023-43465-y)
Supplement: Supplementary file 1 — Supplementary Information [file 41467_2023_43465_MOESM1_ESM.pdf]

Supplementary Information For

Nanoparticles Exhibiting Virus-Mimic Surface Topology for Enhanced Oral Delivery

Zhentao Sang[1,2,8], Lu Xu[3,8], Renyu Ding[4,8], Minjun Wang[1], Xiaoran Yang[1], Xitan Li[4], Bingxin Zhou[1], Kaijun Gou[5], Yang Han[6], Tingting Liu[7], Xuchun Chen[7], Ying Cheng[7*], Huazhe Yang[2*], Heran Li[1*]

[1] School of Pharmacy, China Medical University, Shenyang 110122, China

[2] School of Intelligent Medicine, China Medical University, Shenyang, 110122, China

[3] School of Pharmacy, Shenyang Pharmaceutical University, Shenyang, 110016, China

[4] Department of Intensive Care Unit, The First Hospital of China Medical University, Shenyang 110001, China

[5] Institute of Tibetan Plateau, Southwest Minzu University, Chengdu, 610225, China

[6] Department of Pathology, The First Hospital of China Medical University, Shenyang 110001, China

[7] Department of Organ Transplantation and Hepatobiliary, The First Hospital of China Medical University, Shenyang 110001, China

[8] Zhentao Sang, Lu Xu and Renyu Ding contributed equally

* Correspondence:

Ying Cheng, Department of Organ Transplantation and Hepatobiliary, The First Hospital of China Medical University, 110001 Shenyang, China; E-mail: chengying75@sina.com. Tel.:+8613940392982

Huazhe Yang, School of Intelligent Medicine, China Medical University, 77 Puhe Road, 110122, Shenyang, China; E-mail: hzyang@cmu.edu.cn. Tel.: +8618900911486.

Heran Li, School of Pharmacy, China Medical University, 77 Puhe Road, 110122, Shenyang, China; E-mail: liheranmm@163.com. Tel.: +8613897945866.

Supplementary Methods

Synthesis of fluorescent NPs. SSN, MSN, VSN and CVSN were labeled with fluorescent tags RITC or FITC as depicted in our previous reports[1]. Briefly, 100 mg RITC or FITC was dissolved in 2 ml ethanol under light avoidance. Then 100 μl APTES was added and stirred for 24 h to prepare an ethanol solution of RITC-APTES or FITC-APTES. For in vitro and in vivo trace, NPs was dispersed in 2 ml ethanol followed by the addition of 100 μl RITC-APTES. After stirring for 24 h, the system was centrifuged for separation, washed with ethanol until the supernatant was colorless and dried to obtain RITC or FITC labeled NPs.

Stability of fluorescent NPs. fluorescent NPs were incubated in SGF, SIF and SBF at 37 ℃ and 100 rpm. At 2, 6 and 12 h post incubation, the fluorescent spectra of samples were recorded by a microplate reader (SpectraMax M3, Molecular-Devices, US). At the end of incubation (24 h), the samples were separated and the supernatant were sent to fluorescence detection.

In vitro degradation property of NPs. Degradation property of NPs was determined using weight loss method. Briefly, 10 mg NPs (n=3) were incubated with 2 ml SGF, SIF and SBF, respectively at 37 ℃ and 100 rpm. At the preset time points, the samples were separated by centrifugation, washed with water, thoroughly dried, weighed, and calculated the degradation rate. After that, the degradation process was repeated until the samples completely degraded.

In vitro biocompatibility of NPs. First, the cytotoxicity of NPs was determined by cell counting kit-8 (CCK-8) assay. Briefly, Caco-2 cells were first seeded in 96-wells plates at a density of 10^4 cells/well and incubated under 5% CO_2 at 37 ℃ for 24 h. Then the cells were incubated with the serial dilutions of SSN, MSN, VSN and CVSN (n=3) for 24-72 h. After adding CCK-8 solution (10 μl) to each well and incubating for 4 h, the absorbance was measured in a microplate reader at 450 nm.

Next, protein adsorption and hemolysis tests were performed to disclose the blood compatibility of NPs. 10 mg NPs (n=3) was evenly dispersed in 5 ml SBF under ultrasound. Then 5 ml BSA solution (0.5 mg/ml) was added and stirred at 37 ℃ for 6 h. After centrifugation, the supernatant was filtered, and the concentration of BSA was measured by ultraviolet-visible spectroscopy (UV-vis, UV-1750, Shimadzu, Japan) at 280 nm. The adsorption rate was calculated as:

$$BSA\ Adsorption\ Rate\ (\%) = \frac{(C_0 - C_\mathrm{n})\ V}{m} \times 100\% \qquad (1)$$

Where C_0 and C_n stand for the initial concentration and the endpoint concentration, respectively.

For the hemolysis test, orbital venous blood of SD rats was collected and placed in a heparin-coated tube. After centrifuging, the red blood cells (RBCs) in the bottom layer were collected, cleaned with sterile normal saline for three times, and diluted into 2% (v/v) RBCs suspension with sterile normal saline. Then the RBCs suspensions were mixed with the normal saline suspensions of NPs in equal volume to prepare the mixtures with concentrations of 5, 10, 20, 50 and 100 μg/ml, respectively. In particular, normal saline and deionized water were mixed with 2% RBCs suspension in equal volume and served as negative control and positive control, respectively. The suspensions were kept in a sterile incubator at 37 ℃ for 4 h, and centrifuged at 612 g for 5 min. The absorbance of the supernatant was measured at 540 nm via UV-vis, and the hemolysis rate was calculated

according to the formula:

$$Hemolysis\ Rate\ (\%) = \frac{A_T - A_N}{A_P - A_N} \times 100\% \qquad (2)$$

Where A_T represents the absorbance value of the tested group, A_N and A_P are the absorbance value of positive and negative controls, respectively.

In vivo toxicity of NPs. ICR mice were employed to evaluate the biosafety of the NPs, which were fed adaptively for 1 week after arrival, divided into 5 groups (including control group and SSN, MSN, VSN and CVSN groups, n=3), and oral administrated with normal saline, or the normal saline suspension of SSN, MSN, VSN and CVSN at the dose of 200 mg/kg, respectively. The environmental temperature, humidity and light were strictly controlled during the whole experiment. The body weight and clinic manifestations were recorded every alternate day. At the 14th day, mice were fasted overnight in advance, and the orbital vein blood was collected and sent to the hematology analysis (COBAS, INTEGRA 800, RoChe, Germany) and the blood biochemical analysis (pocH-100i, SYSMEX, Japan), respectively. Afterwards, the rats were euthanized and the organs were excised, weighted, fixed in formalin, embedded in paraffin, sectioned, dewaxed, and stained with H&E for histological studies.

Supplementary Figures

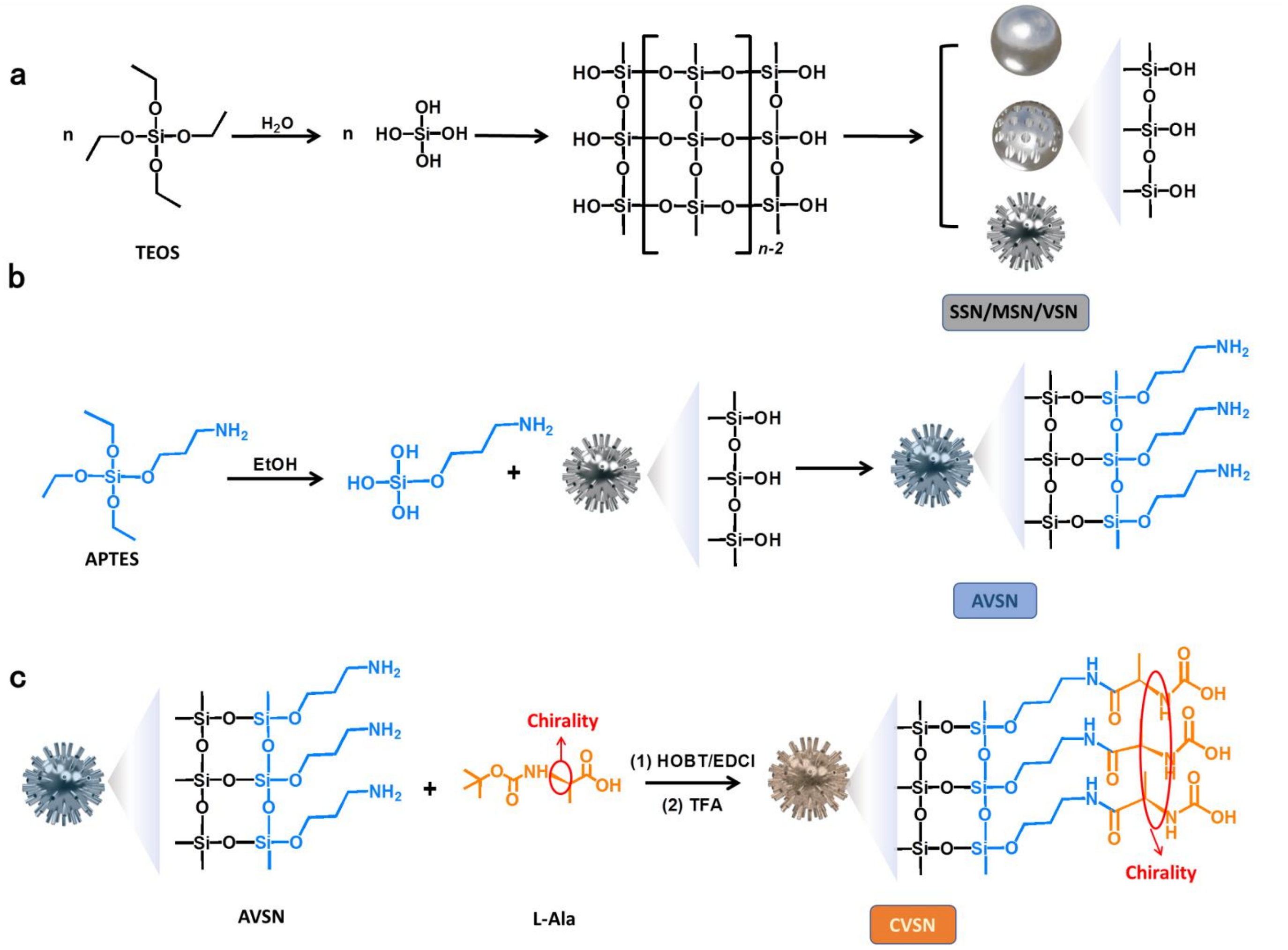

Supplementary Figure 1. Synthetic routes of NPs. The molecular synthetic strategy for **a** SSN/MSN/VSN**, b** AVSN and **c** CVSN**.**

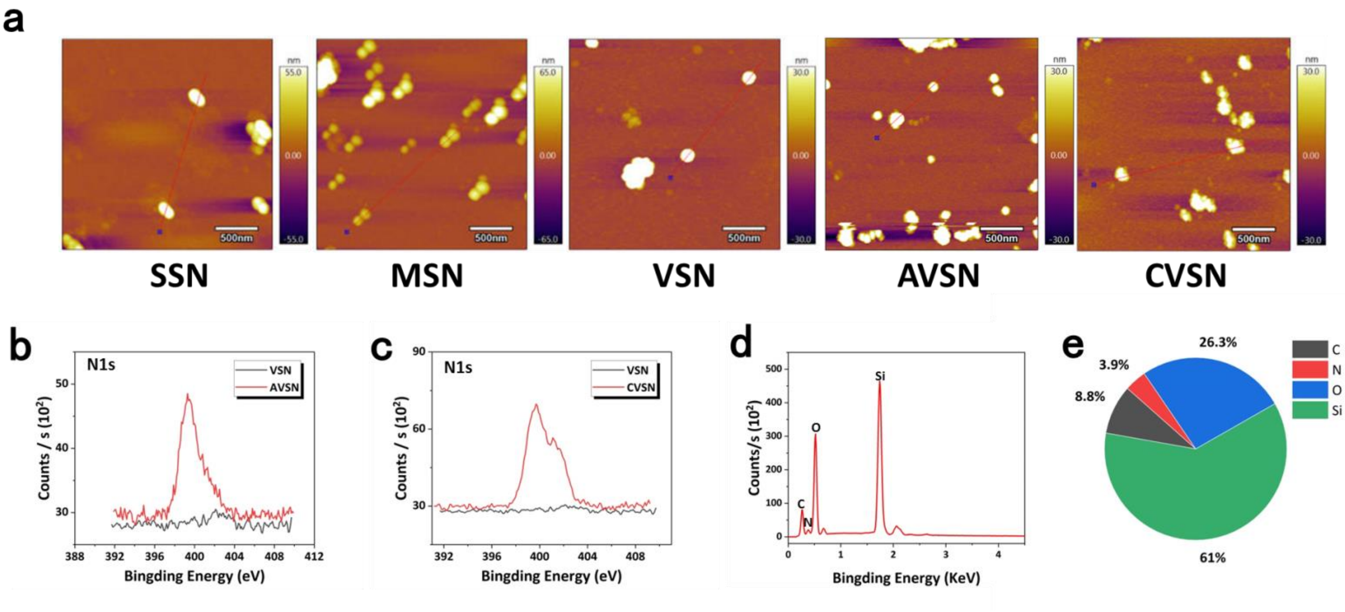

Supplementary Figure 2. structure confirmation of NPs. a Two-dimensional (2D) AFM images of NPs. **b, c** XPS spectra of VSN, AVSN and CVSN. **d** X-ray energy spectrum (EDS) analysis and **e** element ratio (e) of CVSN. Source data of **b-e** are provided as a Source Data file.

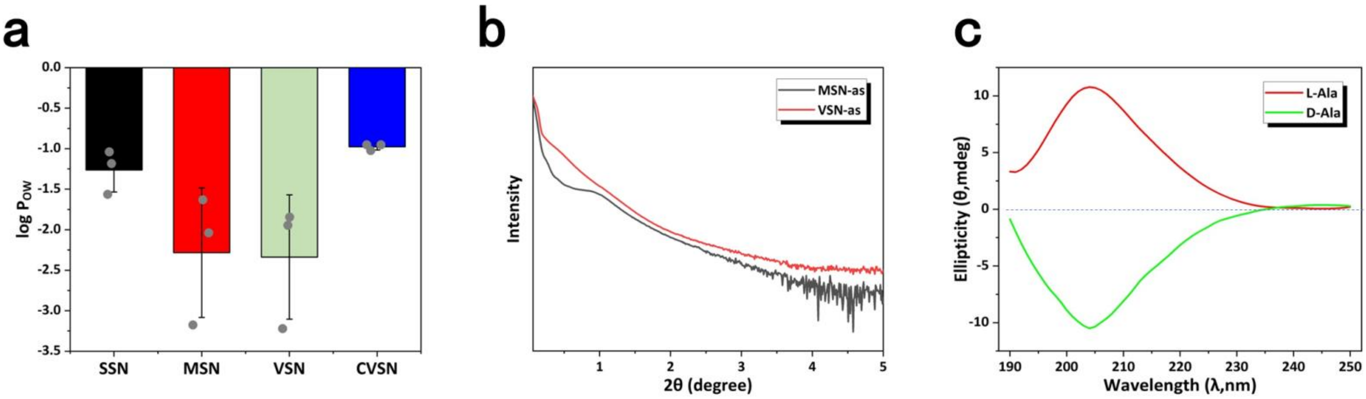

Supplementary Figure 3. Surface interface property of NPs. a P_{ow} value of NPs. Data are presented as the mean ± SD (n = 3 independent experiments). **b** SAXD patterns of MSN-as and VSN-as before the removal of CTAB templates. **c** CD spectra of L/D-Ala. Source data of **a-c** are provided as a Source Data file.

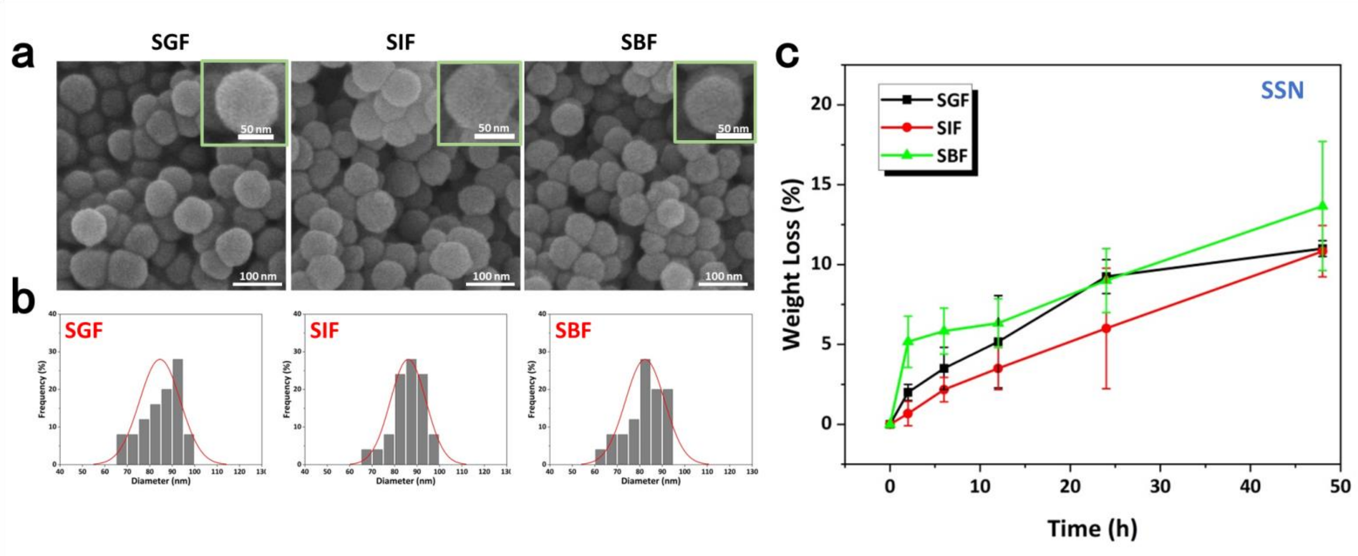

Supplementary Figure 4. Stability of SSN. a Representative SEM images of SSN in SGF, SIF and SBF for 48 h. **b** Particle size distribution of SSN in SGF, SIF and SBF for 48 h. Source data are provided as a Source Data file. **c** Degradation profiles of SSN in SGF, SIF and SBF for 48 h. Data are presented as the mean ± SD (n = 3 independent experiments). Source data are provided as a Source Data file.

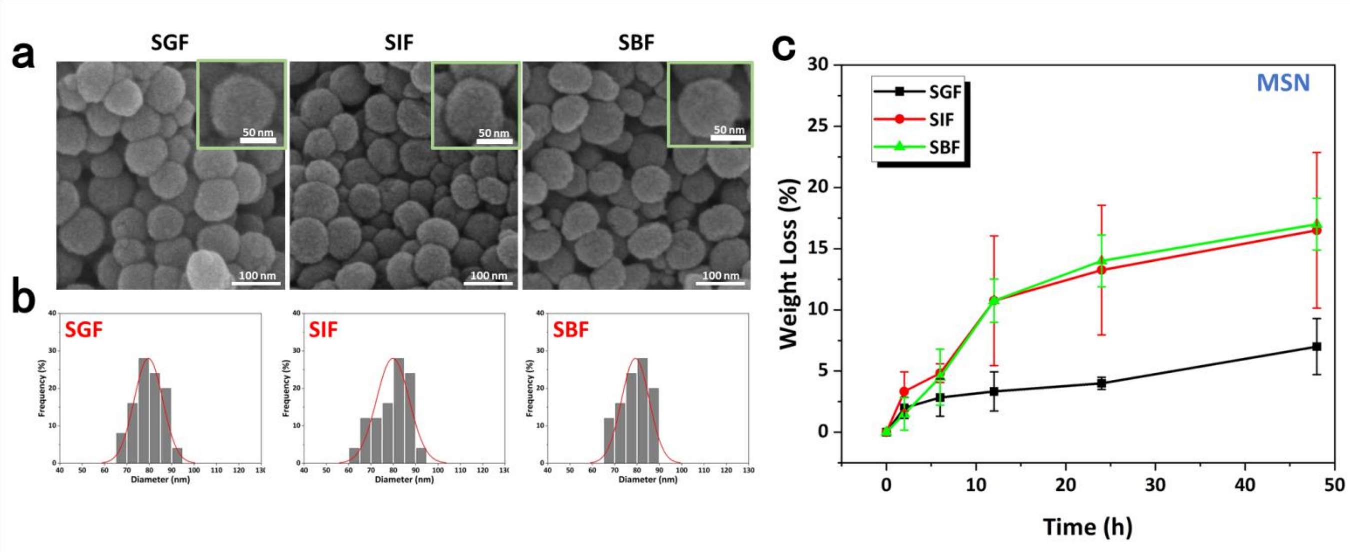

Supplementary Figure 5. Stability of MSN. a Representative SEM images of MSN in SGF, SIF and SBF for 48 h. **b** Particle size distribution of MSN in SGF, SIF and SBF for 48 h. Source data are provided as a Source Data file. **c** Degradation profiles of MSN in SGF, SIF and SBF for 48 h. Data are presented as the mean ± SD (n = 3 independent experiments). Source data are provided as a Source Data file.

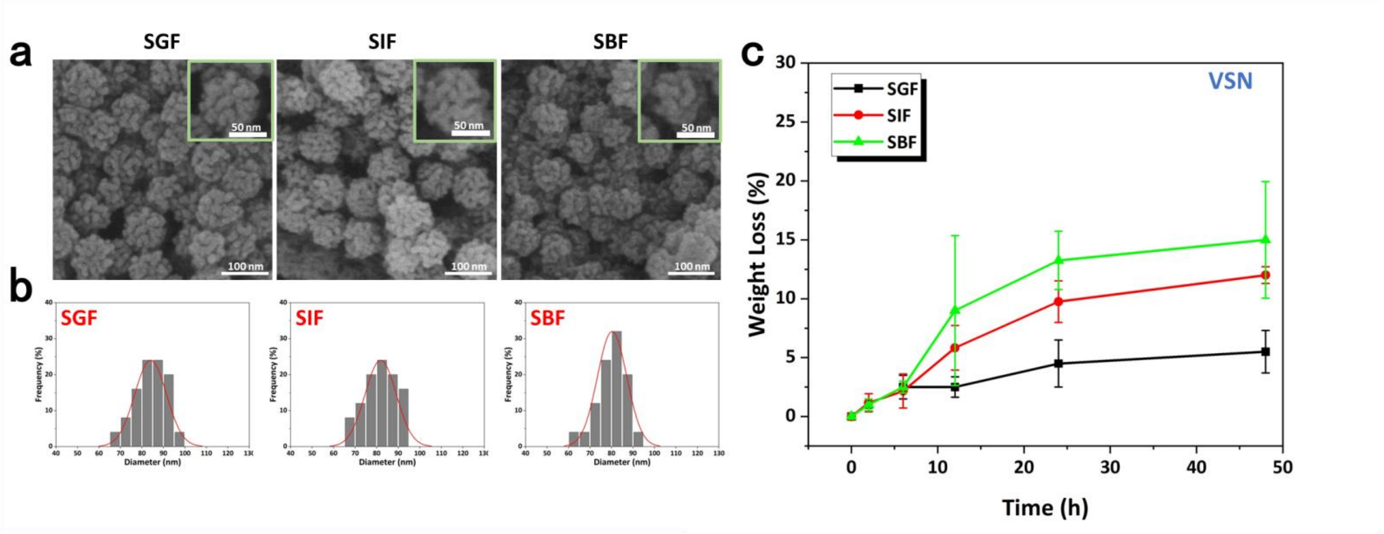

Supplementary Figure 6. Stability of VSN. a Representative SEM images of VSN in SGF, SIF and SBF for 48 h. **b** Particle size distribution of VSN in SGF, SIF and SBF for 48 h. Source data are provided as a Source Data file. **c** Degradation profiles of VSN in SGF, SIF and SBF for 48 h. Data are presented as the mean ± SD (n = 3 independent experiments). Source data are provided as a Source Data file.

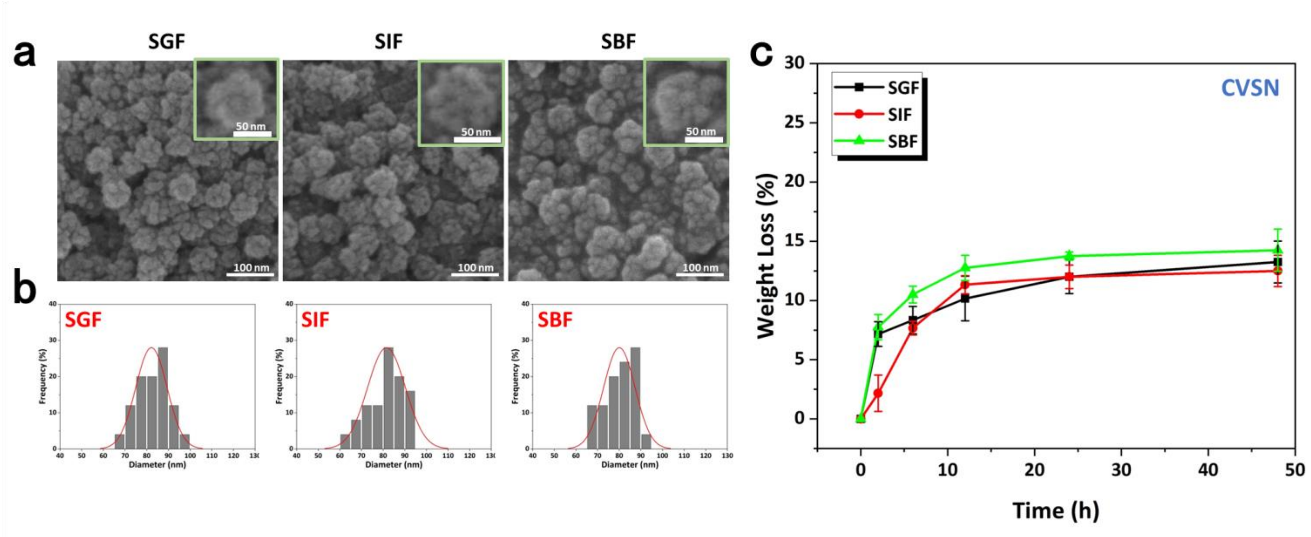

Supplementary Figure 7. Stability of CVSN. a Representative SEM images of CVSN in SGF, SIF and SBF for 48 h. **b** Particle size distribution of CVSN in SGF, SIF and SBF for 48 h. Source data are provided as a Source Data file. **c** Degradation profiles of CVSN in SGF, SIF and SBF for 48 h. Data are presented as the mean ± SD (n = 3 independent experiments). Source data are provided as a Source Data file.

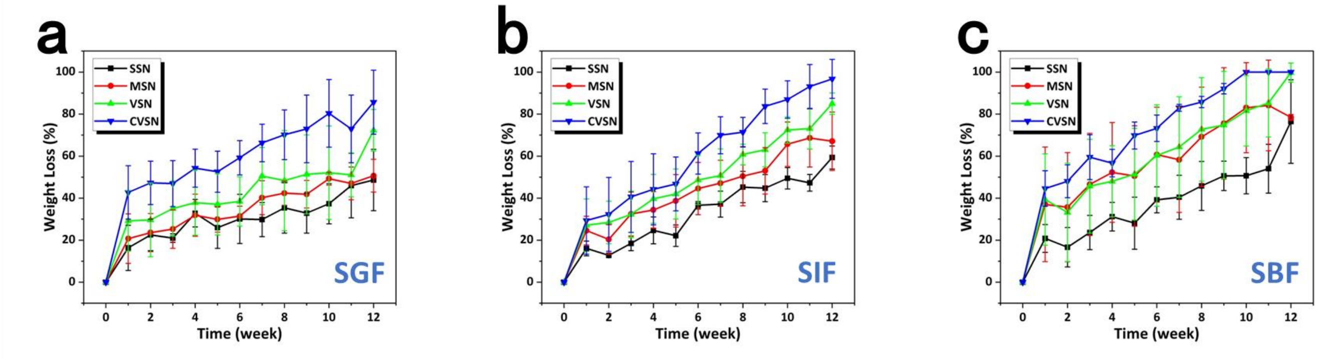

Supplementary Figure 8. Degradation tendency of NPs. Degradation profiles of NPs in **a** SGF, **b** SIF and **c** SBF for 12 weeks. Data are presented as the mean ± SD (n = 3 independent experiments). Source data of a-c are provided as a Source Data file.

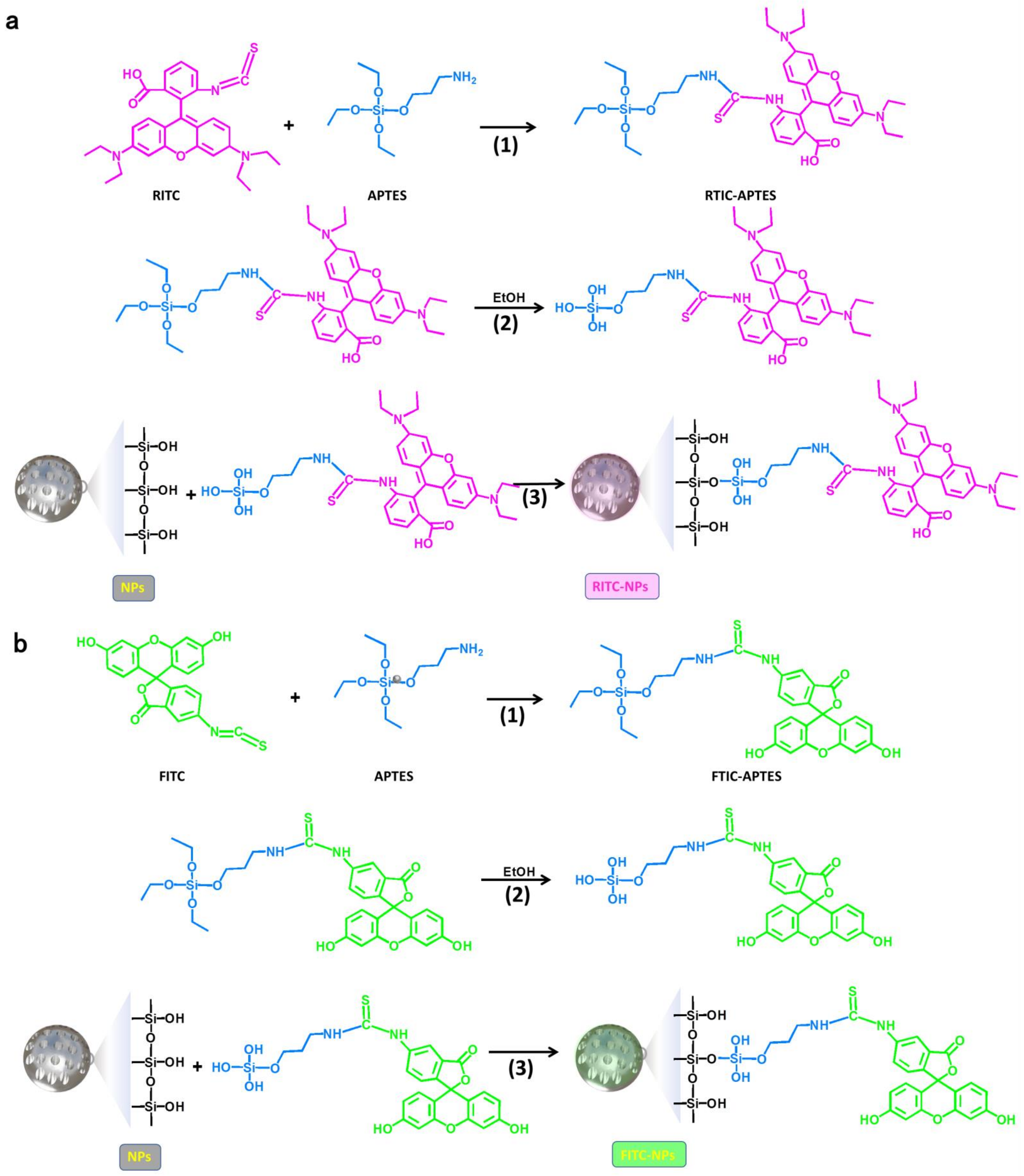

Supplementary Figure 9. Synthetic routes of Fluorescent NPs. The molecular synthetic strategy for **a** RITC labeled NPs and **b** FITC labeled NPs.

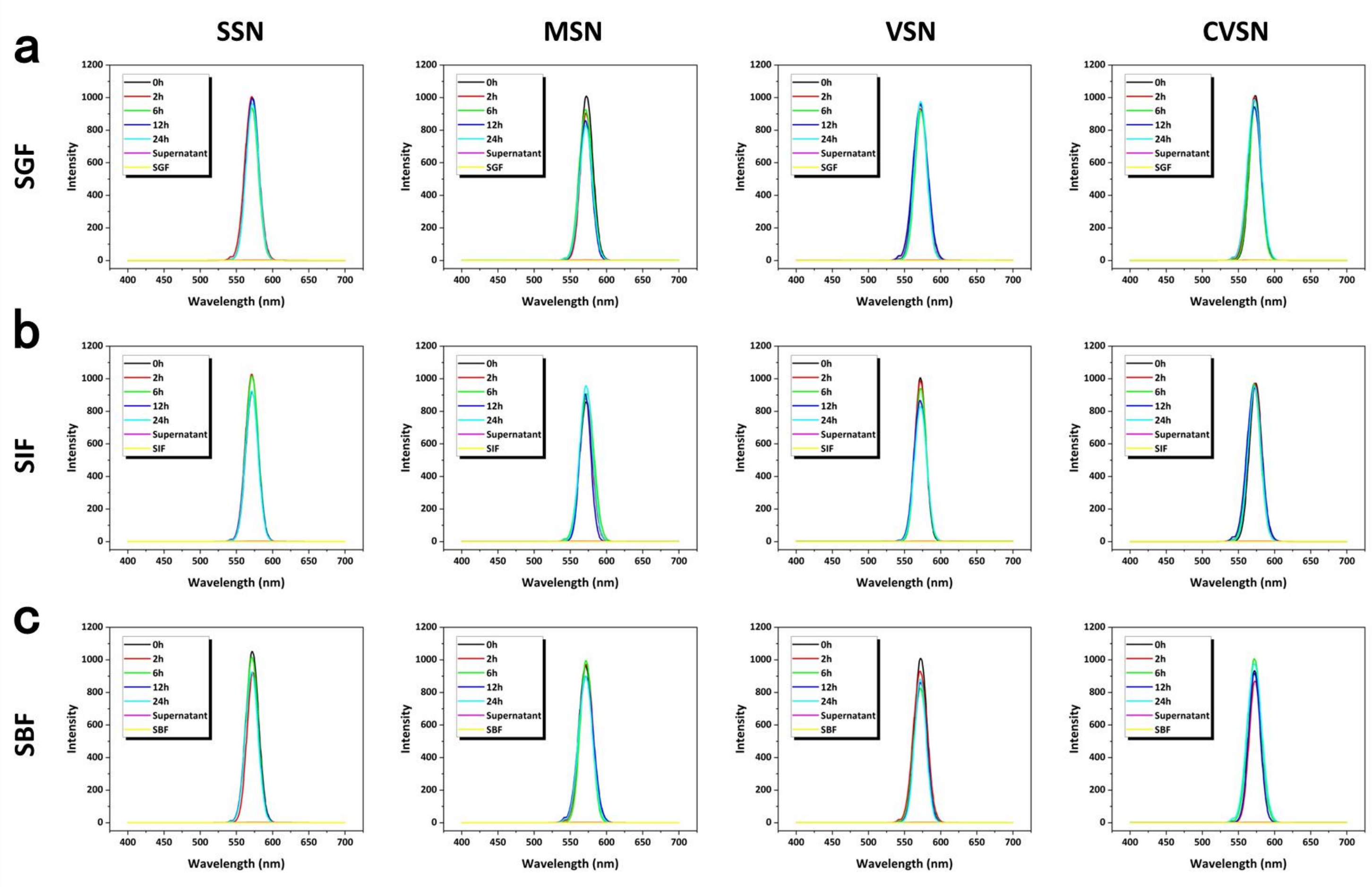

Supplementary Figure 10. Stability of fluorescent NPs. Fluorescent spectra of RITC labeled NPs after incubating in **a** SGF, **b** SIF and **c** SBF for different period. Source data are provided as a Source Data file.

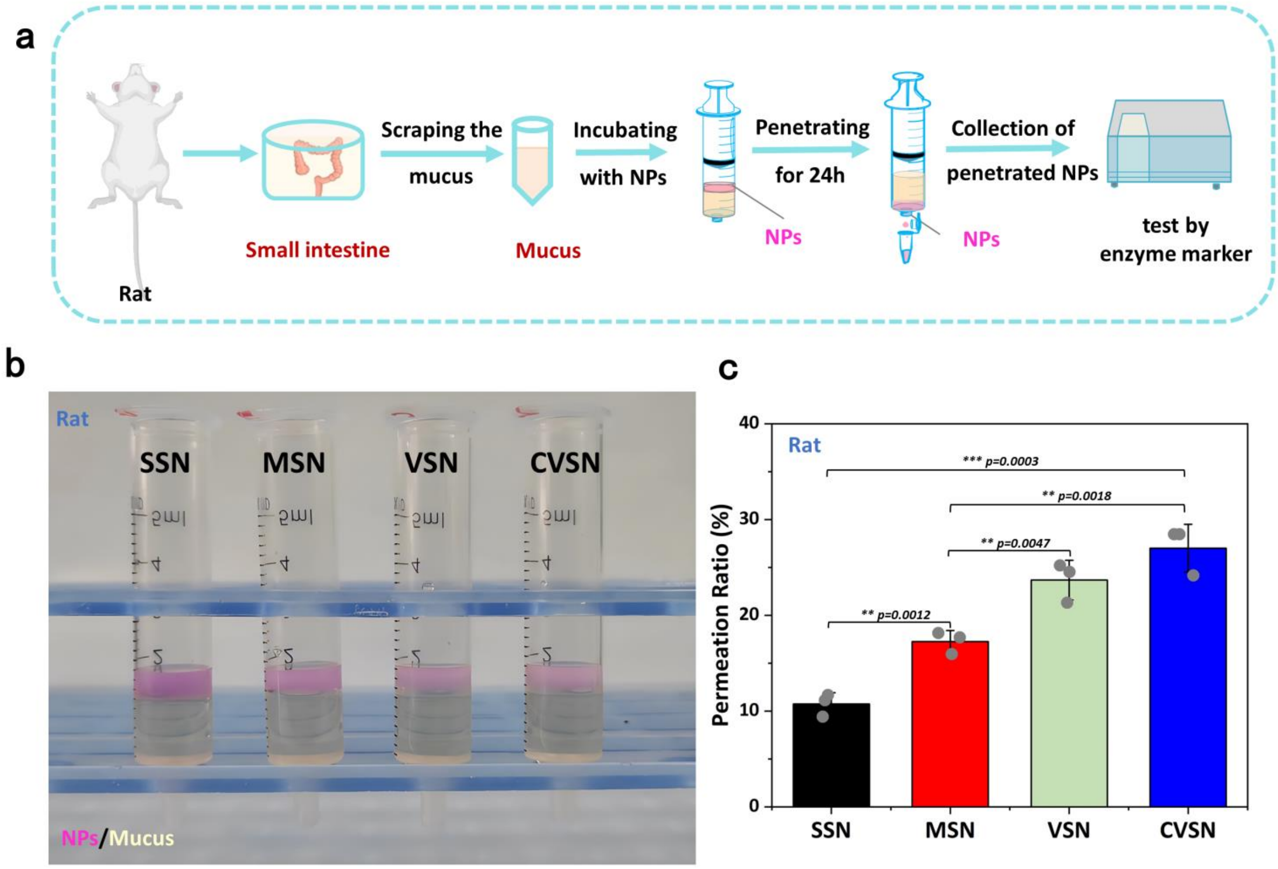

Supplementary Figure 11. Permeation of NPs in the rat intestinal mucus. a Treatment protocols on the permeation of NPs in the rat intestinal mucus. **b** Images and **c** permeation ratio of the permeation of NPs on rat intestinal mucus. Data are presented as the mean ± SD (n = 3 independent experiments). **$P < 0.01$ and ***$P < 0.001$ by two-tailed Student's t-test. Source data are provided as a Source Data file.

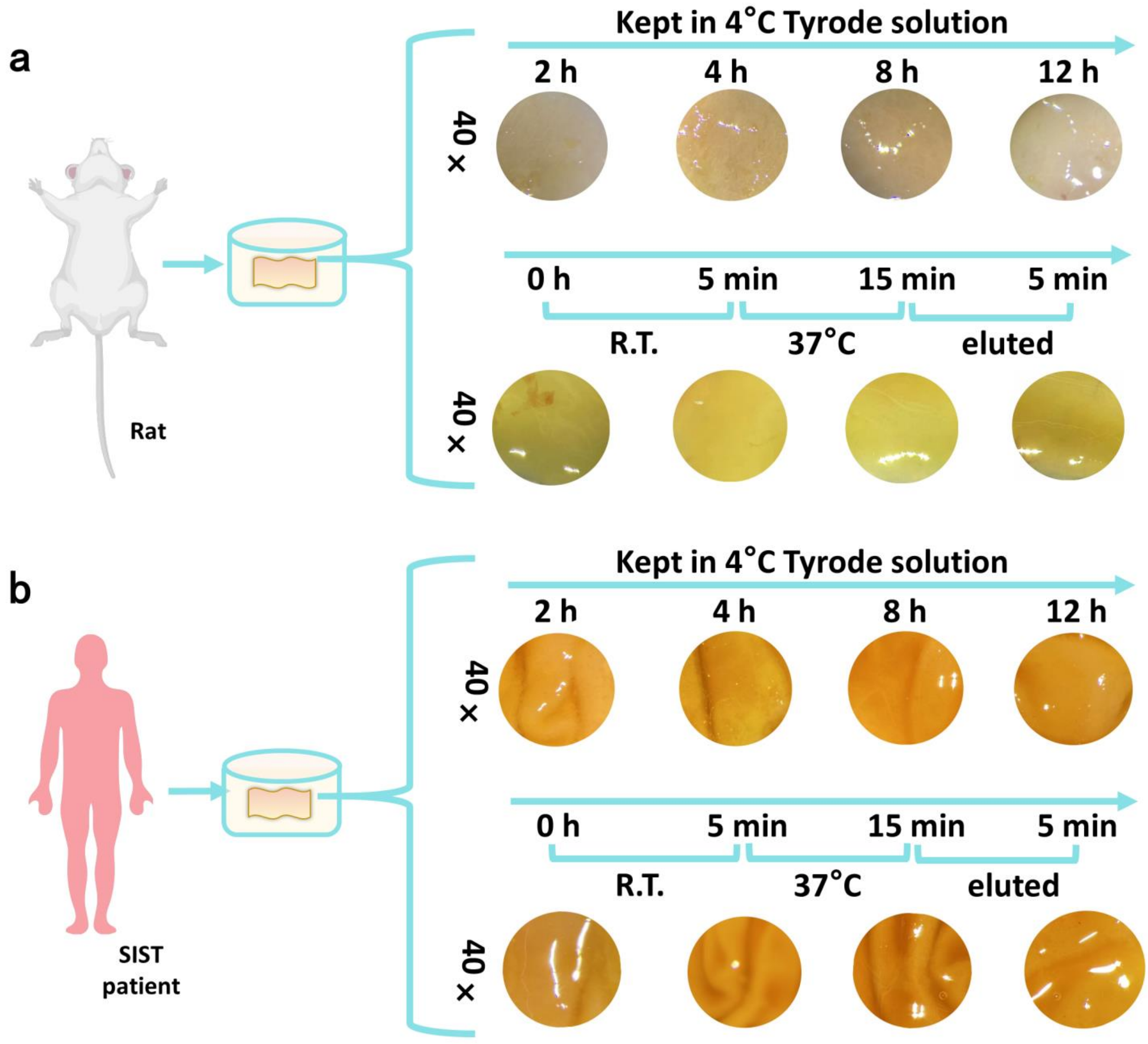

Supplementary Figure 12. Intestinal mucus barrier integrity during bio-adhesion study. Microscope images on the intestinal mucus of **a** rat and **b** human after 12 h storage and the experimental procedure. Experiment was repeated three times independently with similar results.

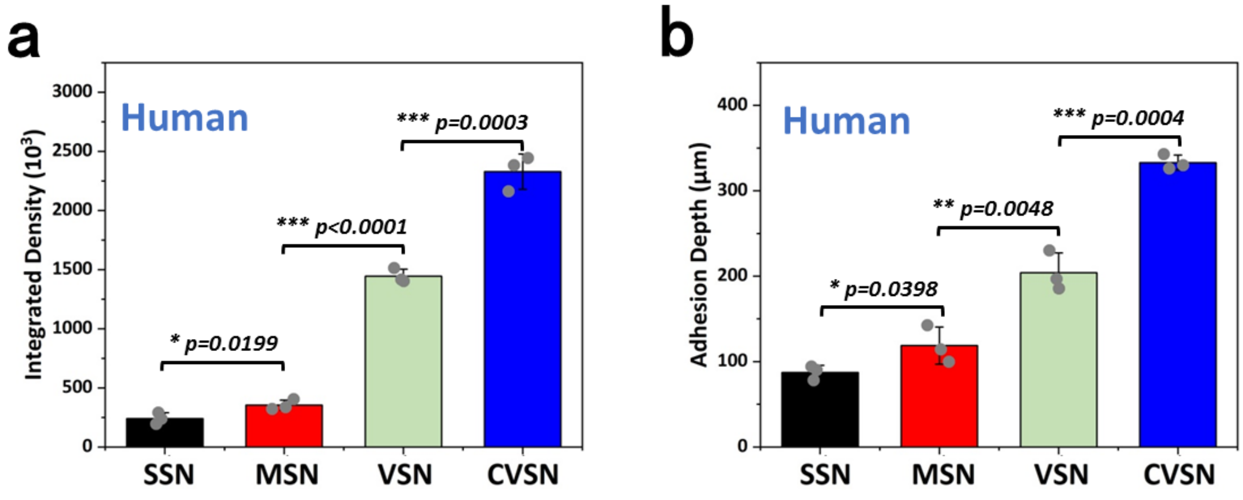

Supplementary Figure 13. Bio-adhesion of NPs on human intestinal mucosa. a Fluorescence integrated density and **b** permeation depth of NPs on human intestinal mucosa. Data are presented as the mean ± SD (n = 3 independent experiments). *P < 0.05, **P < 0.01 and ***P < 0.001 by two-tailed Student's t-test. Source data are provided as a Source Data file.

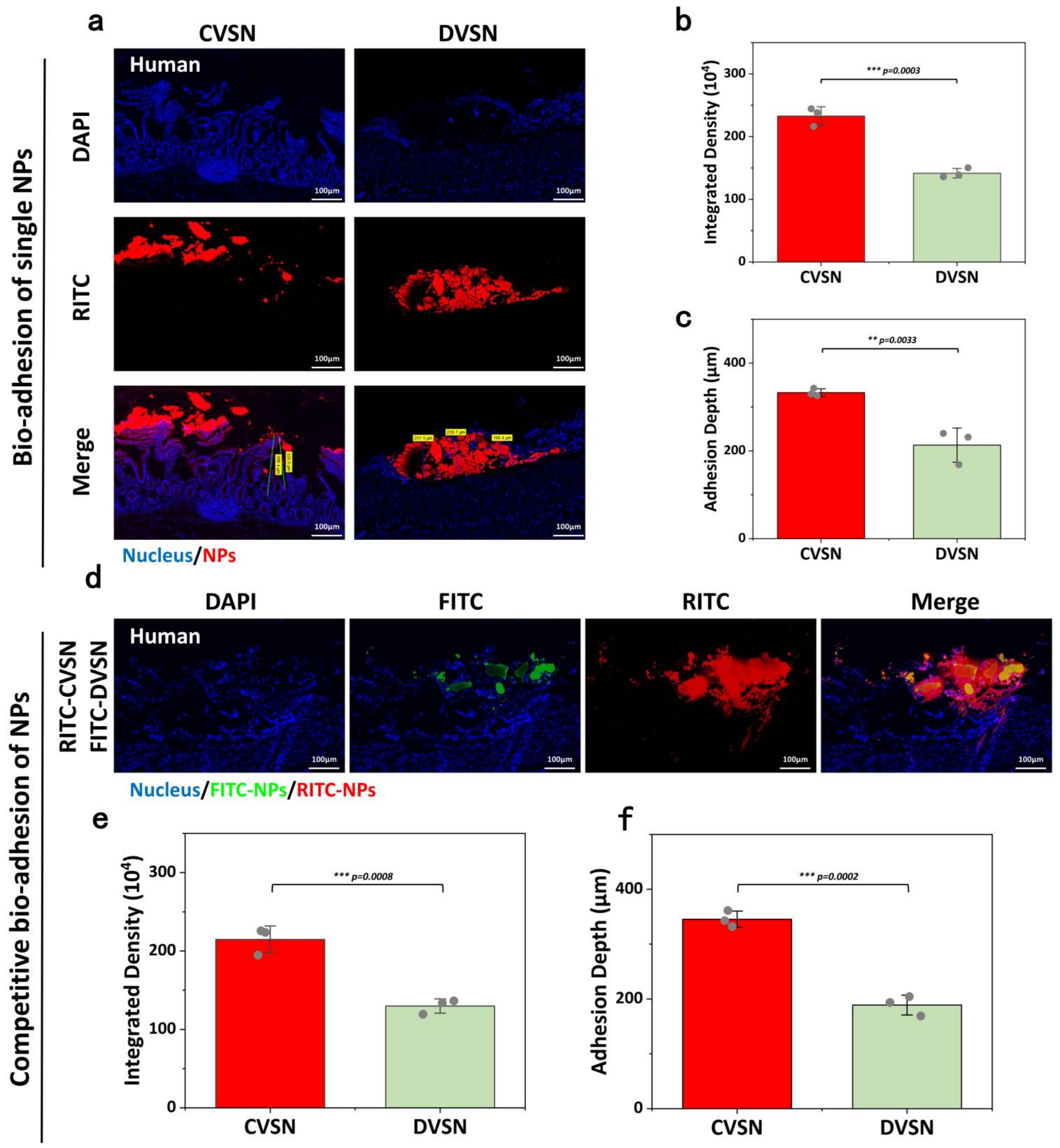

Supplementary Figure 14. Comparison on the bio-adhesion of NPs with different chirality. a. CLSM images of RITC labeled CVSN or DVSN remained on the human intestinal mucosa; blue: nuclei of the intestinal mucosa stained with DAPI, red: RITC labeled NPs, scale bar: 100 μm. Experiment was repeated three times independently with similar results. **b** Fluorescence integrated density and **c** permeation depth of NPs on the human intestinal mucosa. Data are presented as the mean ± SD (n = 3 independent experiments). $^{**}P < 0.01$ and $^{***}P < 0.001$ by two-tailed Student's t-test. Source data are provided as a Source Data file. **d** CLSM images of RITC-CVSN and FITC-DVSN competitivly remained on the human intestinal mucosa, blue: nuclei of the

intestinal mucosa stained with DAPI, green: FITC labeled DVSN, red: RITC labeled CVSN. Experiment was repeated three times independently with similar results. **e** Fluorescence integrated density and **f** permeation depth of NPs on the human intestinal mucosa. Data are presented as the mean ± SD (n = 3 independent experiments). ***P < 0.001 by two-tailed Student's t-test. Source data are provided as a Source Data file.

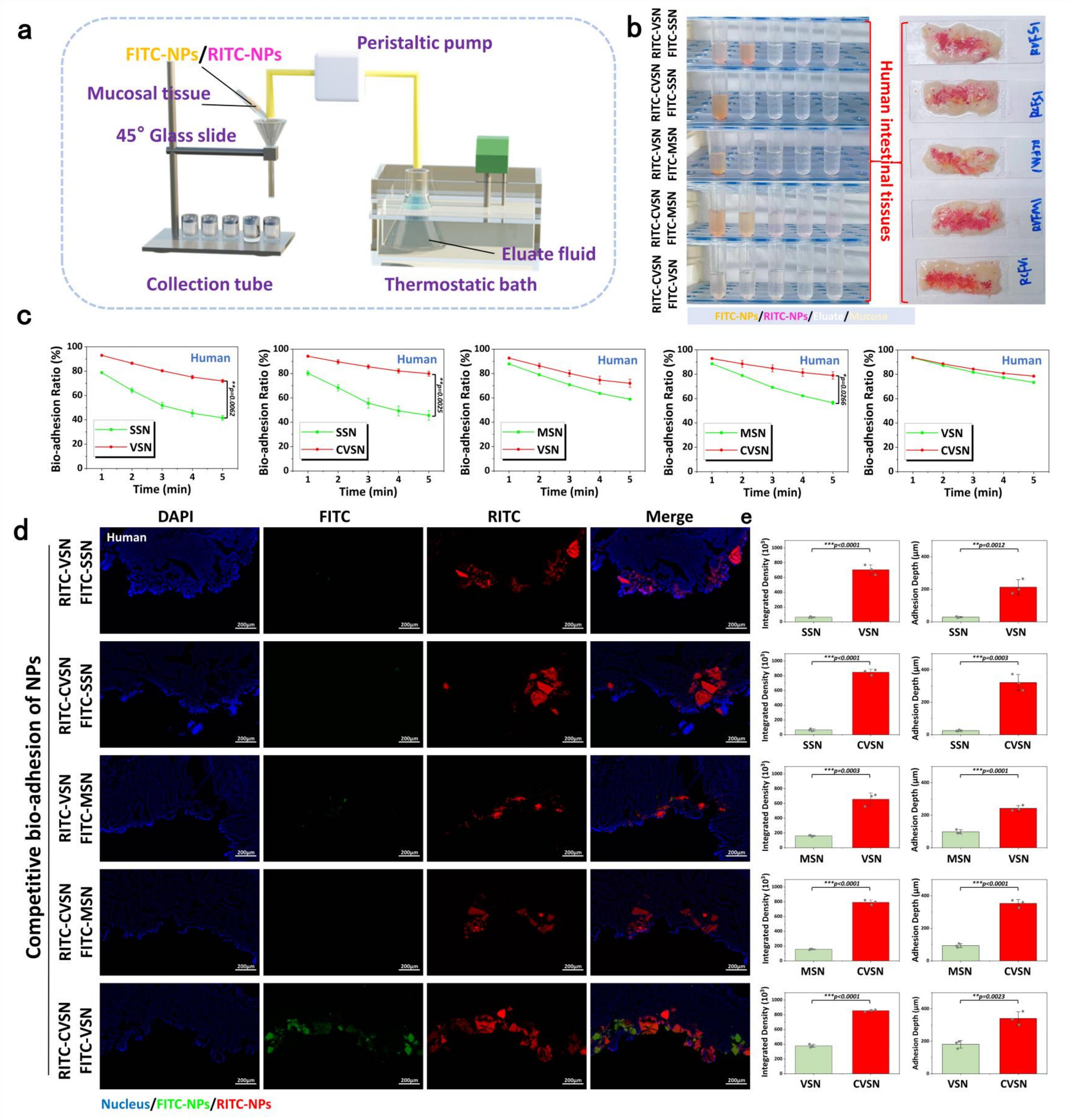

Supplementary Figure 15. Competitive adhesion of NPs on the human intestinal mucosa. a Schematic

illustration of the apparatus on competitive bio-adhesion study. **b** The competitive bio-adhesion images of RITC/FITC labeled NPs performed on the human intestinal mucosa. Experiment was repeated three times independently with similar results.**c** The competitive bio-adhesion profiles of NPs on the human intestinal mucosa. Data are presented as the mean ± SD (n = 3 independent experiments). *P < 0.05 and **P < 0.01 by two-tailed Student's t-test. Source data are provided as a Source Data file. **d** CLSM images of different fluorescent labeled NPs competitivly remained on the human intestinal mucosa; blue: nuclei of the intestinal mucosa stained with DAPI, green: FITC labeled NPs, red: RITC labeled NPs. Experiment was repeated three times independently with similar results. **e** Fluorescence integrated density and permeation depth of NPs on the human intestinal mucosa. Data are presented as the mean ± SD (n = 3 independent experiments). **P < 0.01 and ***P < 0.001 by two-tailed Student's t-test. Source data are provided as a Source Data file.

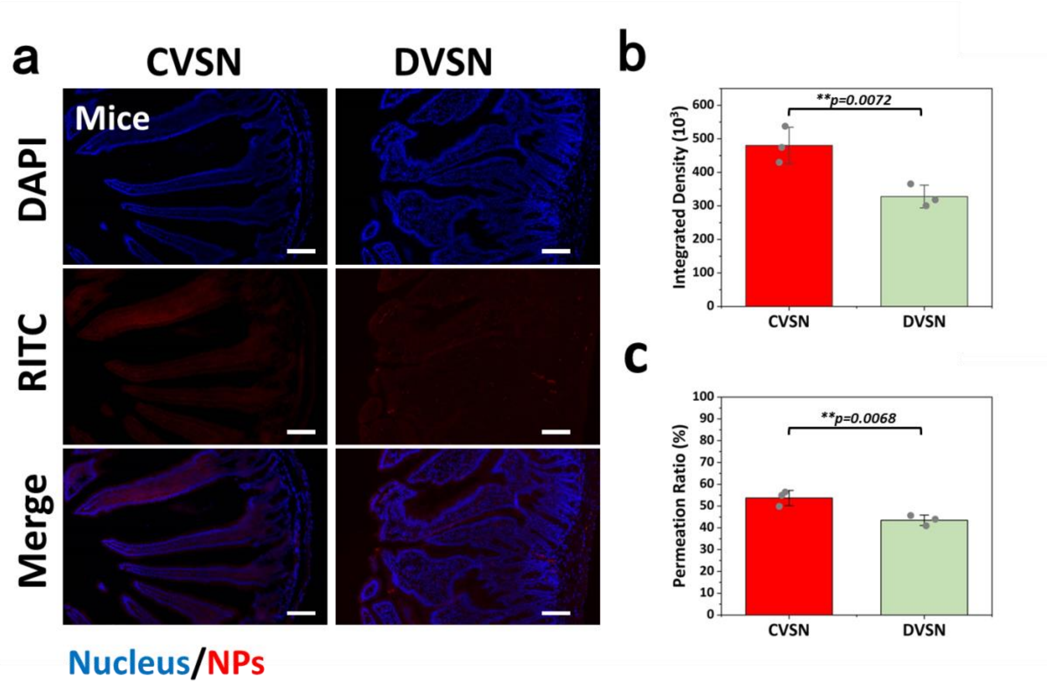

Supplementary Figure 16. Comparison on the adsorption of NPs with different chirality through the intestinal villi. a CLSM images of CVSN and DVSN absorbed at the small intestinal villi of mice at 2 h post oral administration; blue: nuclei of the intestinal villi stained with DAPI, red: RITC labeled NPs, scale bar: 100 μm. Experiment was repeated three times independently with similar results. **b** Fluorescence integrated density and **c** permeation ratio of NPs at the small intestinal villi of mice. Data are presented as the mean ± SD (n = 3 independent experiments). **P < 0.01 by two-tailed Student's t-test. Source data are provided as a Source Data file.

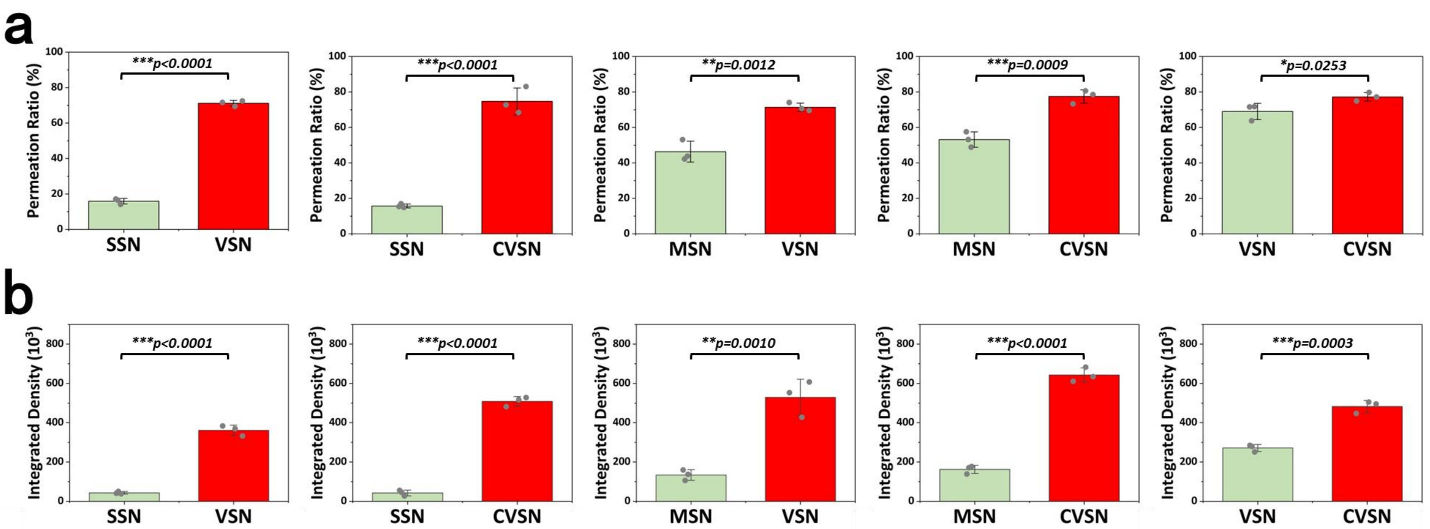

Supplementary Figure 17. Competitive adsorption of NPs in the intestinal villi of mice. a Fluorescence integrated density and **b** permeation ratio of NPs at the small intestinal villi of mice. Data are presented as the mean ± SD (n = 3 independent experiments). **P < 0.01 and ***P < 0.001 by two-tailed Student's t-test. Source data are provided as a Source Data file.

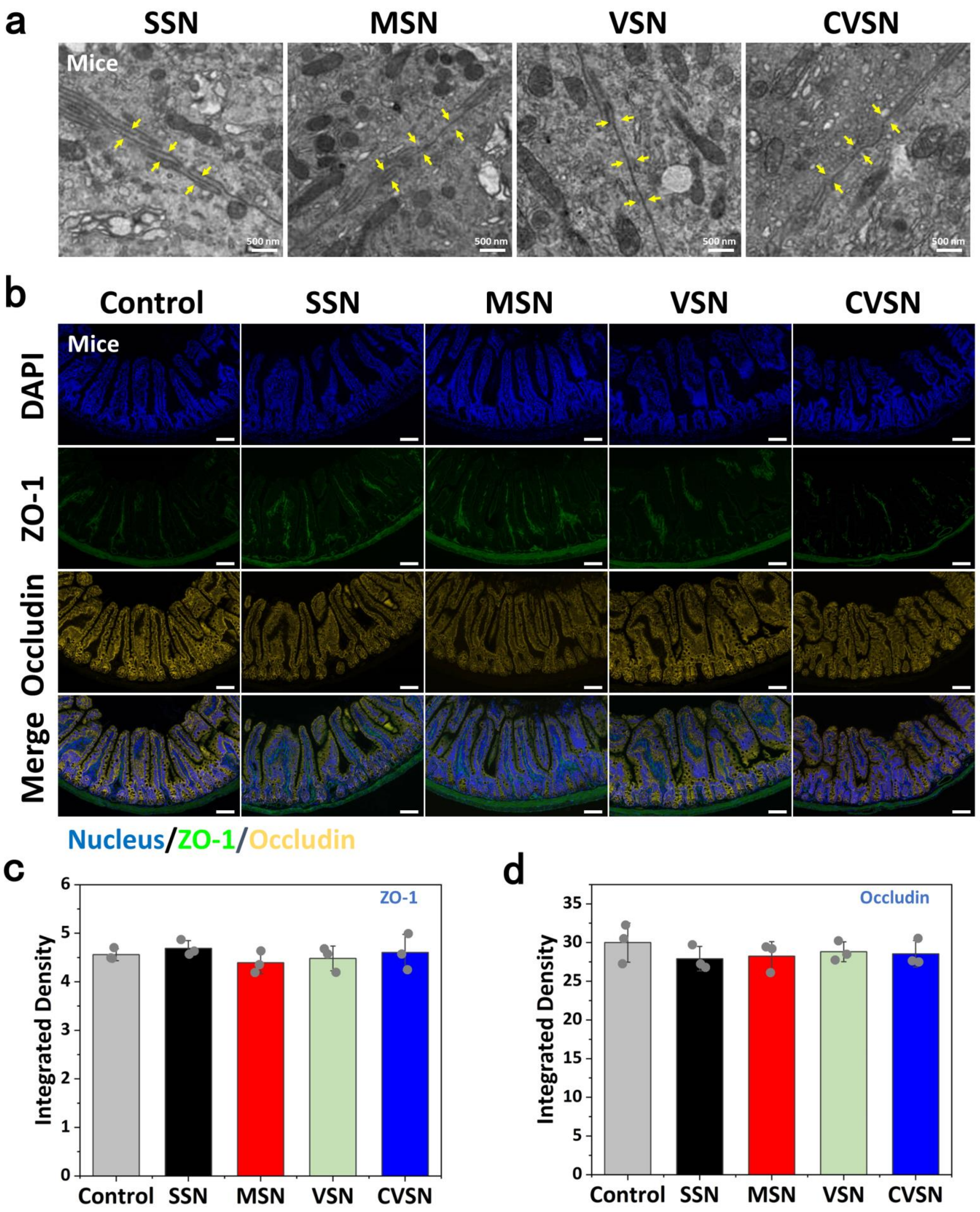

Supplementary Figure 18. Transport of NPs through the tight junction. a Bio-TEM on the tight junctions between epithelial cells of the small intestine of mice at 4 h post oral administration of NPs. Experiment was

repeated three times independently with similar results. **b** Immunofluorescence staining images of the small intestine of mice to indicate the expression of tight junction proteins, including ZO-1 and Occludin; blue: nuclei of the intestinal villi stained with DAPI, green: ZO-1, yellow: Occludin, scale bar: 100 μm. Experiment was repeated three times independently with similar results. Fluorescence integrated density on the expression of **c** ZO-1 and **d** Occludin. Data are presented as the mean ± SD (n = 3 independent experiments). Source data are provided as a Source Data file.

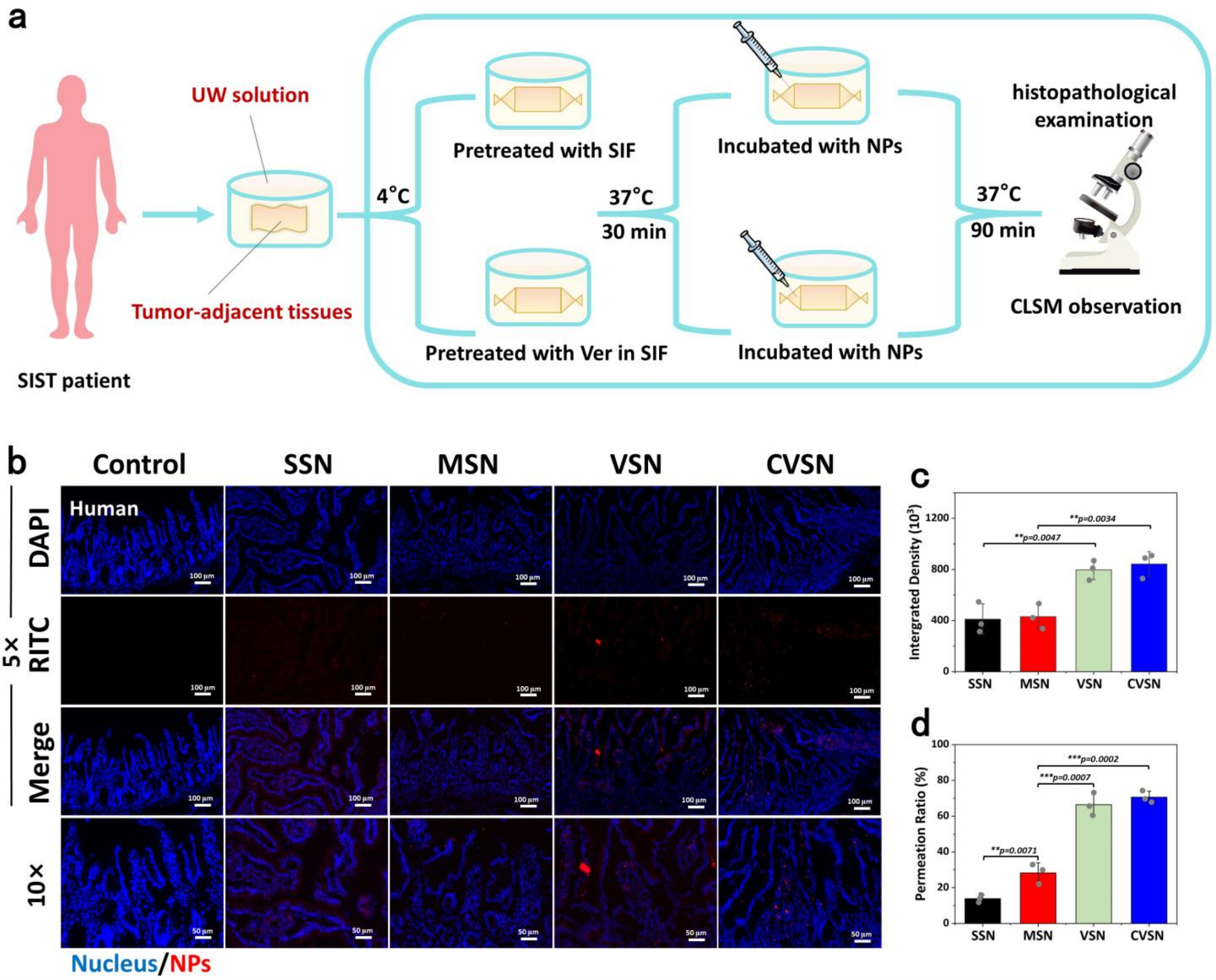

Supplementary Figure 19. NPs absorbed at the ex vivo small intestinal villi of human with the per-treatment of VER. a Treatment protocols on the ex vivo penetration and absorption study pretreated with or without VER on human small intestinal tissues. **b** CLSM images of NPs absorbed at the small intestinal villi of human at 1.5 h post incubation with the pre-treatment of VER for 30 min; blue: nuclei of the intestinal villi stained with DAPI, red: RITC labeled NPs. Experiment was repeated three times independently with similar results. **c** Fluorescence integrated density and **d** permeation ratio of NPs at the small intestinal villi of human.

Data are presented as the mean ± SD (n = 3 independent experiments). **P < 0.01 and ***P < 0.001 by two-tailed Student’s t-test. Source data are provided as a Source Data file.

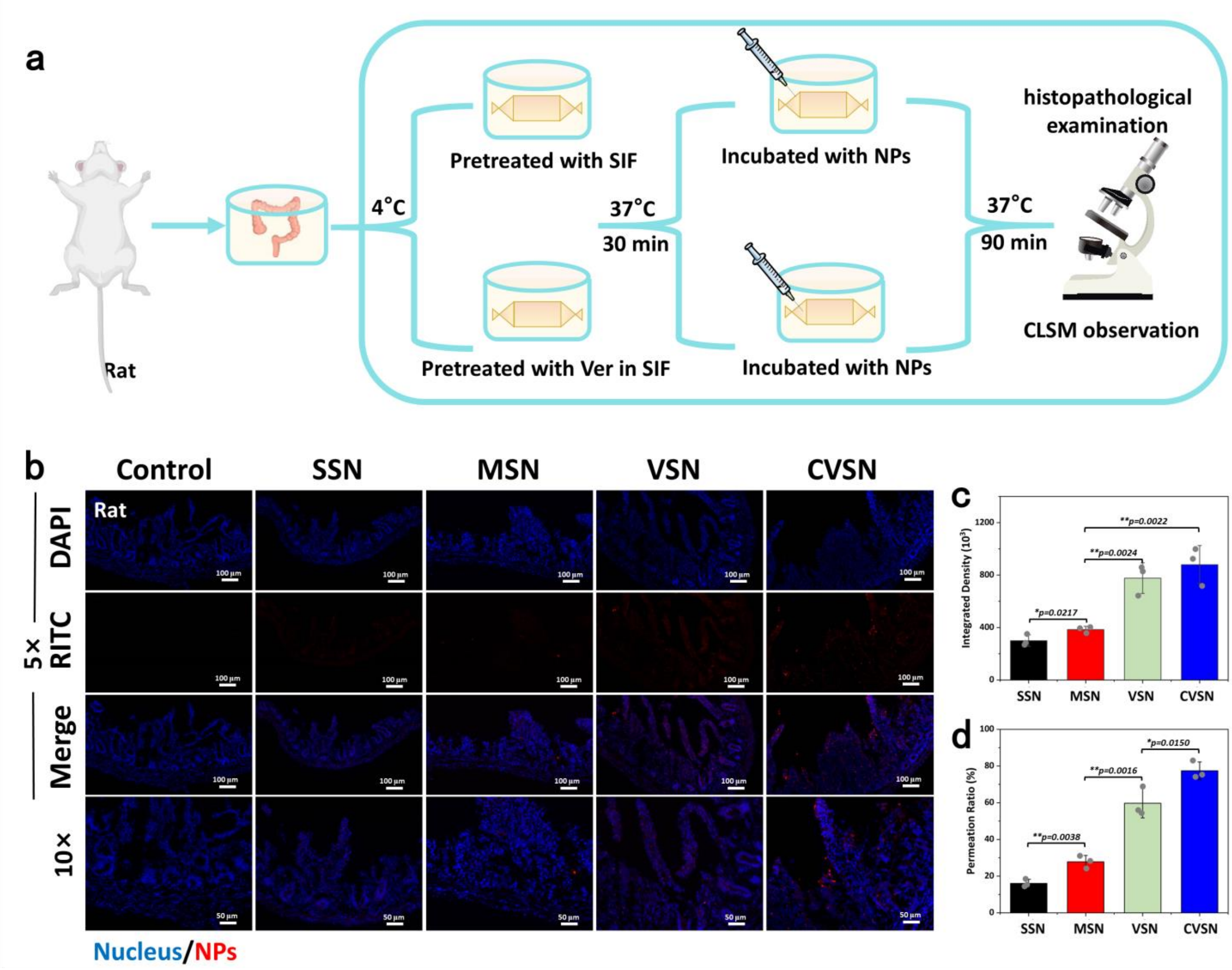

Supplementary Figure 20. NPs absorbed at the ex vivo small intestinal villi of rat without the per-treatment of VER. a Treatment protocols on the ex vivo penetration and absorption study pretreated with or without VER on rat small intestinal tissues. **b** CLSM images of NPs absorbed at the small intestinal villi of rat at 1.5 h post incubation without the pre-treatment of VER; blue: nuclei of the intestinal villi stained with DAPI, red: RITC labeled NPs. Experiment was repeated three times independently with similar results. **c** Fluorescence integrated density and **d** permeation ratio of NPs at the small intestinal villi of rat. Data are presented as the mean ± SD (n = 3 independent experiments). *P < 0.05 and **P < 0.01 by two-tailed Student’s t-test. Source data are provided as a Source Data file.

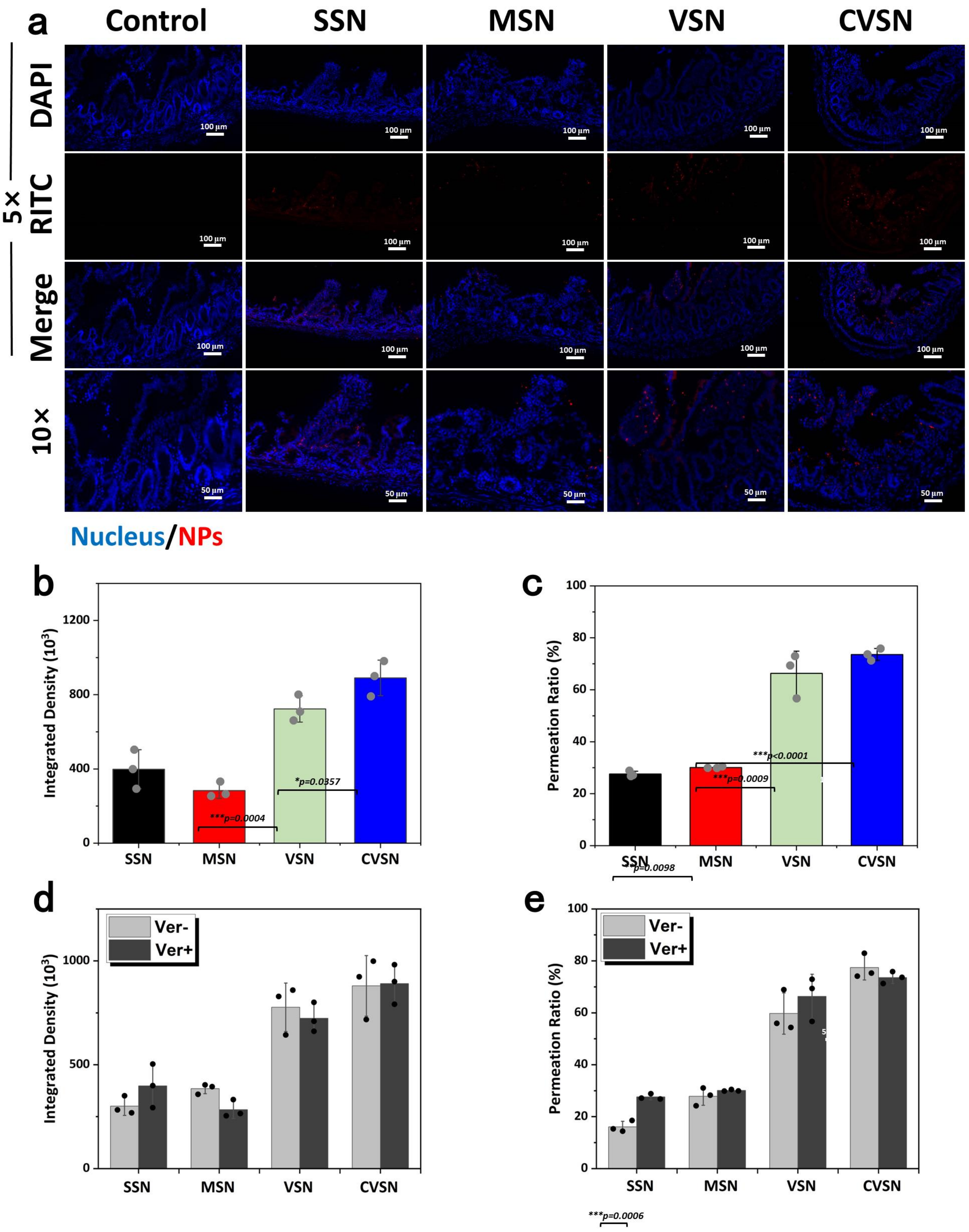

Supplementary Figure 21. NPs absorbed at the ex vivo small intestinal villi of rat with the per-treatment

of VER. a CLSM images of NPs absorbed at the small intestinal villi of rat at 1.5 h post incubation with the pre-treatment of VER for 30 min; blue: nuclei of the intestinal villi stained with DAPI, red: RITC labeled NPs. Experiment was repeated three times independently with similar results. **b** Fluorescence integrated density and **c** permeation ratio of NPs at the small intestinal villi of rats. Data are presented as the mean ± SD (n = 3 independent experiments). *P < 0.05, **P < 0.01 and ***P < 0.001 by two-tailed Student's t-test. Source data are provided as a Source Data file. **d** Fluorescence integrated density and **e** permeation ratio of NPs at the small intestinal villi of rat before and after pretreated with VER. Data are presented as the mean ± SD (n = 3 independent experiments). ***P < 0.001 by two-tailed Student's t-test. Source data are provided as a Source Data file.

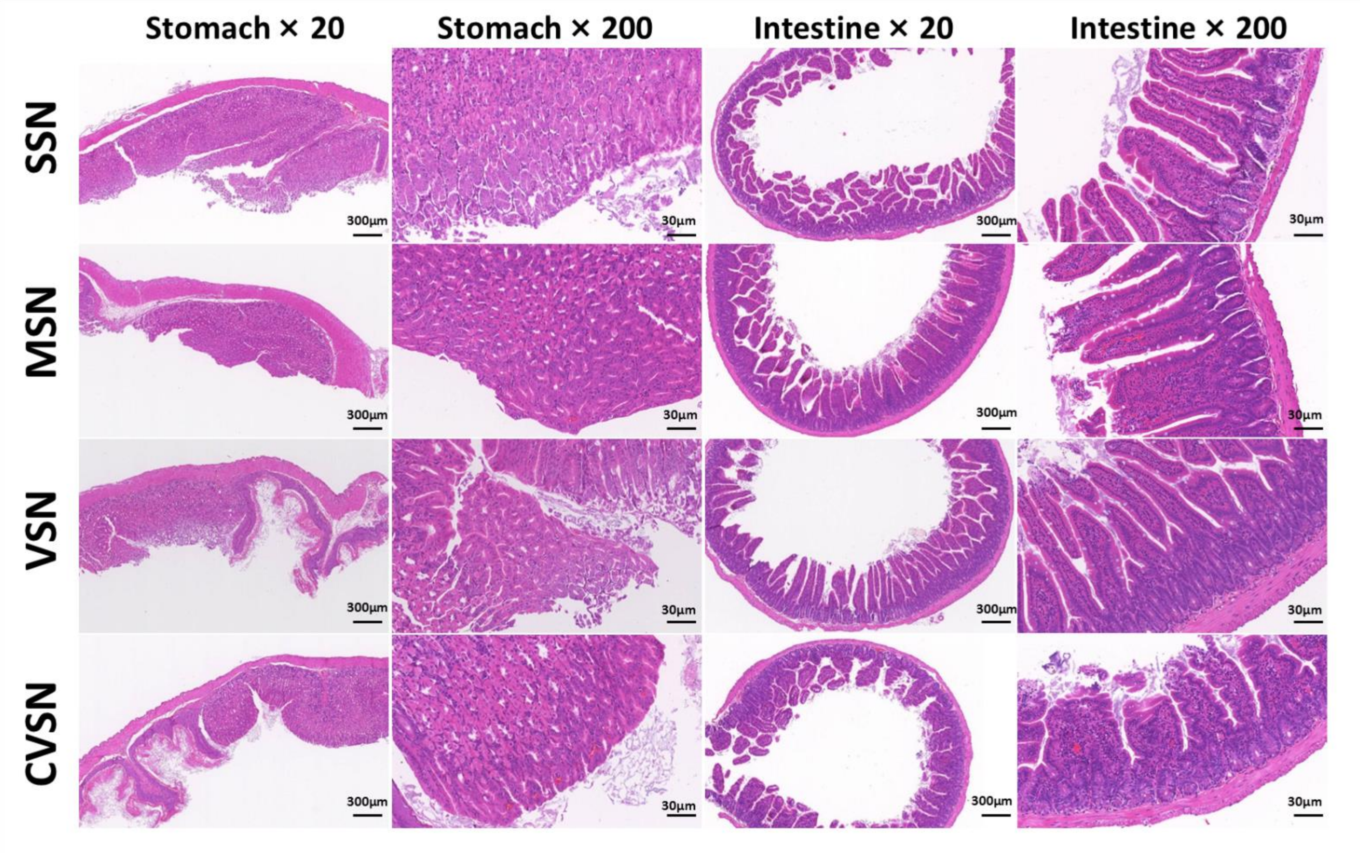

Supplementary Figure 22. GI tract irritation of NPs. Histopathological images of the gastric and small intestinal tissues of mice after oral administration with NPs. Experiment was repeated three times independently with similar results.

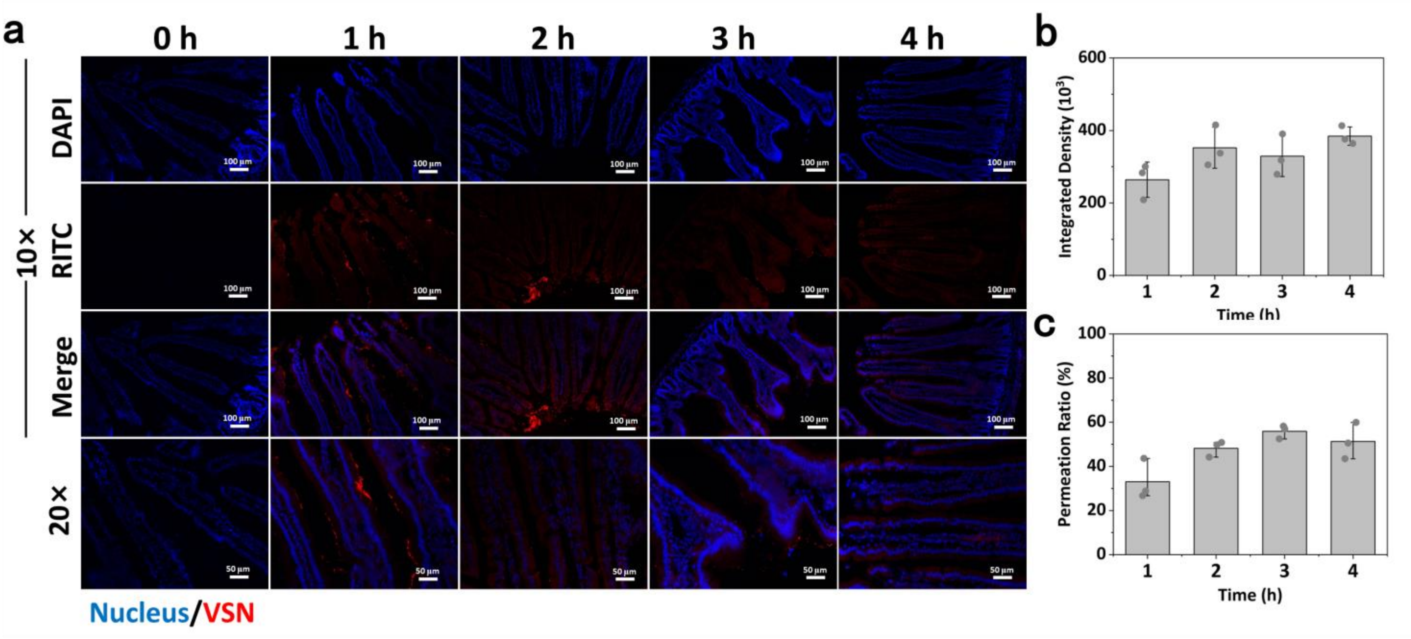

Supplementary Figure 23. Time-dependent absorption of VSN through the intestinal villi. a CLSM images of VSN absorbed at the small intestinal villi of mice at 0, 1, 2, 3 and 4 h post oral administration; blue: nuclei of the intestinal villi stained with DAPI, red: RITC labeled VSN. Experiment was repeated three times independently with similar results. **b** Fluorescence integrated density and **c** permeation ratio of VSN at the small intestinal villi of mice at different times. Data are presented as the mean ± SD (n = 3 independent experiments). Source data are provided as a Source Data file.

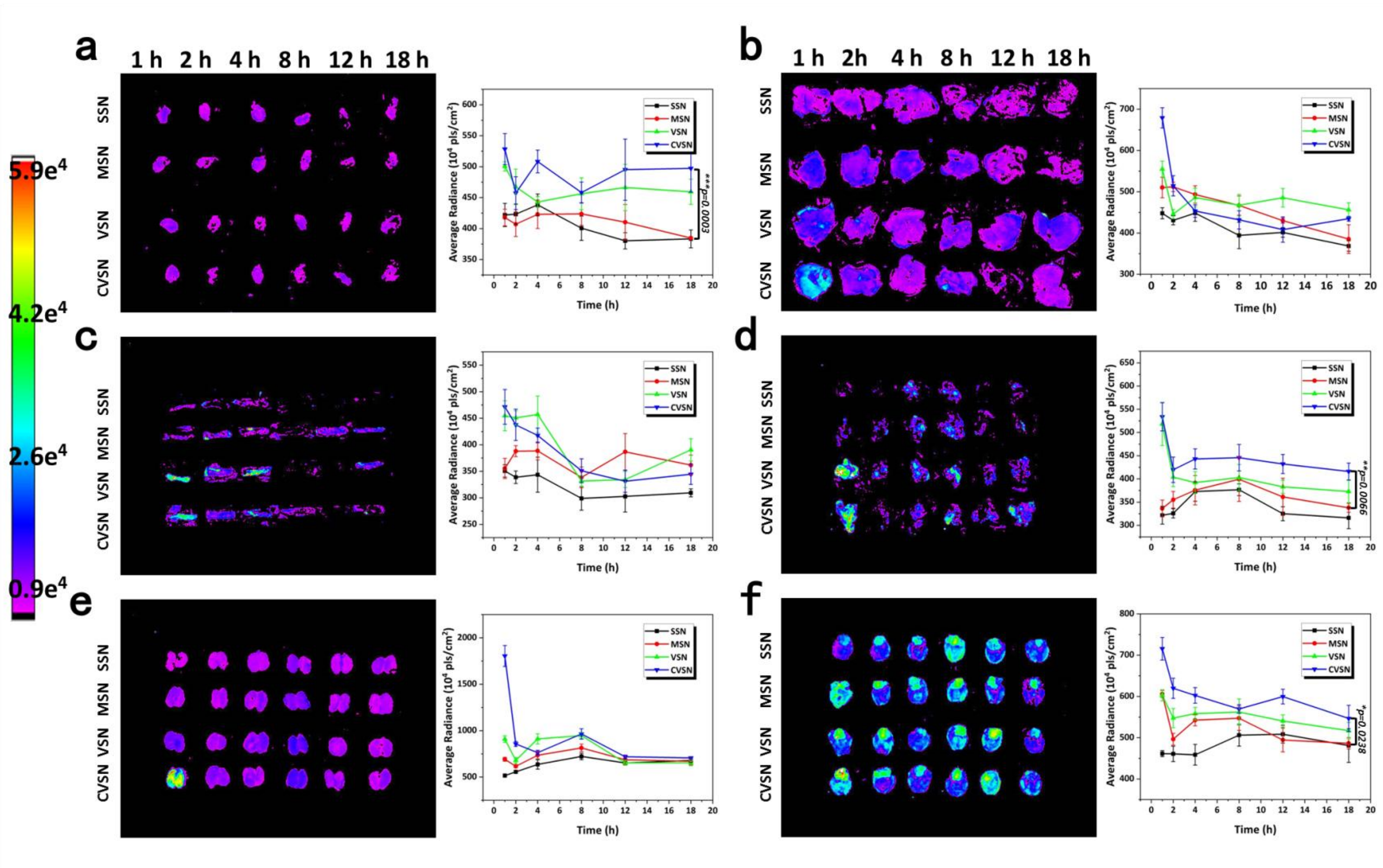

Supplementary Figure 24. Bio-distribution of NPs. Fluorescence images and average accumulation of NPs in the main organs, including **a** heart, **b** liver, **c** spleen, **d** lung, **e** kidney and **f** brain at different times. The color bar indicates the fluorescence intensity. Data are presented as the mean ± SD (n = 3 independent experiments). *P < 0.05 and ***P < 0.001 by two-tailed Student's t-test. Source data are provided as a Source Data file.

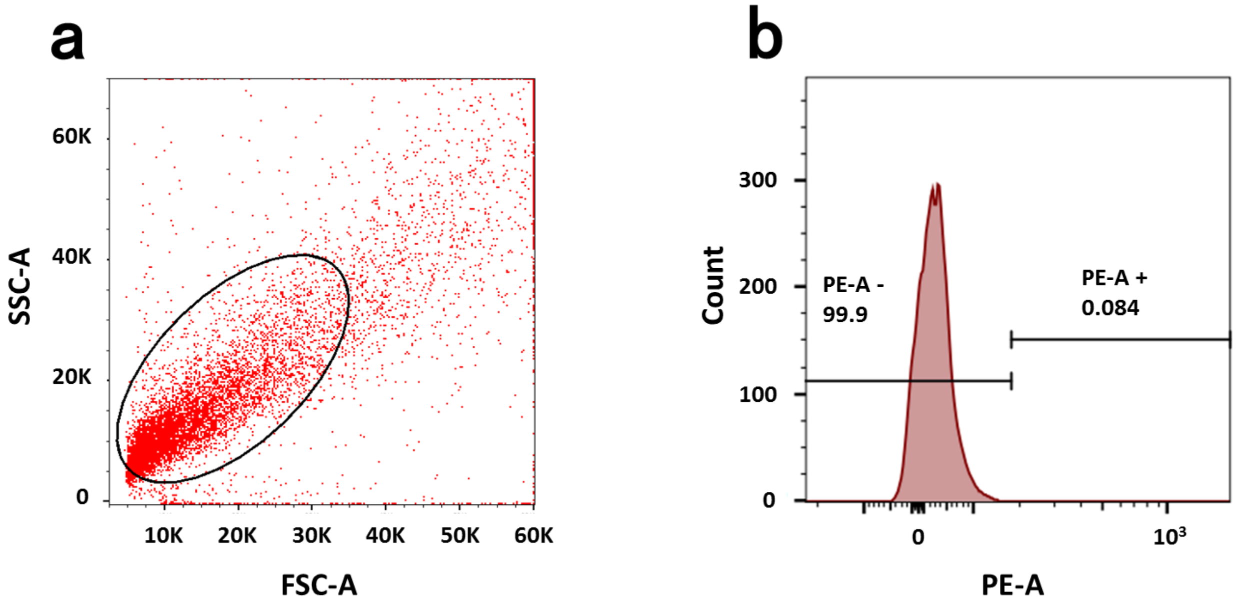

Supplementary Figure 25 Gating strategy for the FCM analysis. a The gating strategy and **b** controlled histogram for cellular internalization of NPs measured via FCM.

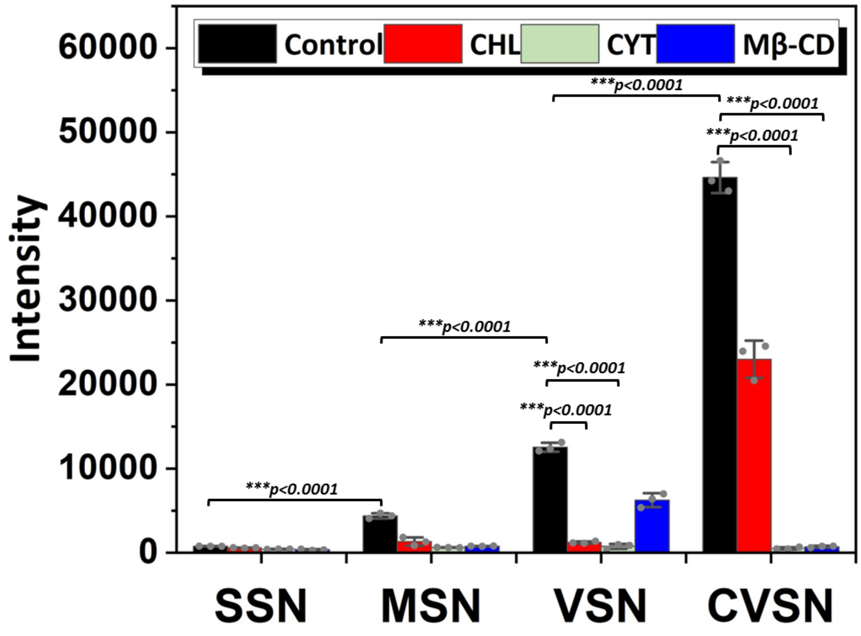

Supplementary Figure 26 Cellular uptake amount of NPs. Cellular internalization efficiency of NPs measured via FCM. Data are presented as the mean ± SD (n = 3 independent experiments). ***P < 0.001 by two-tailed Student's t-test. Source data are provided as a Source Data file.

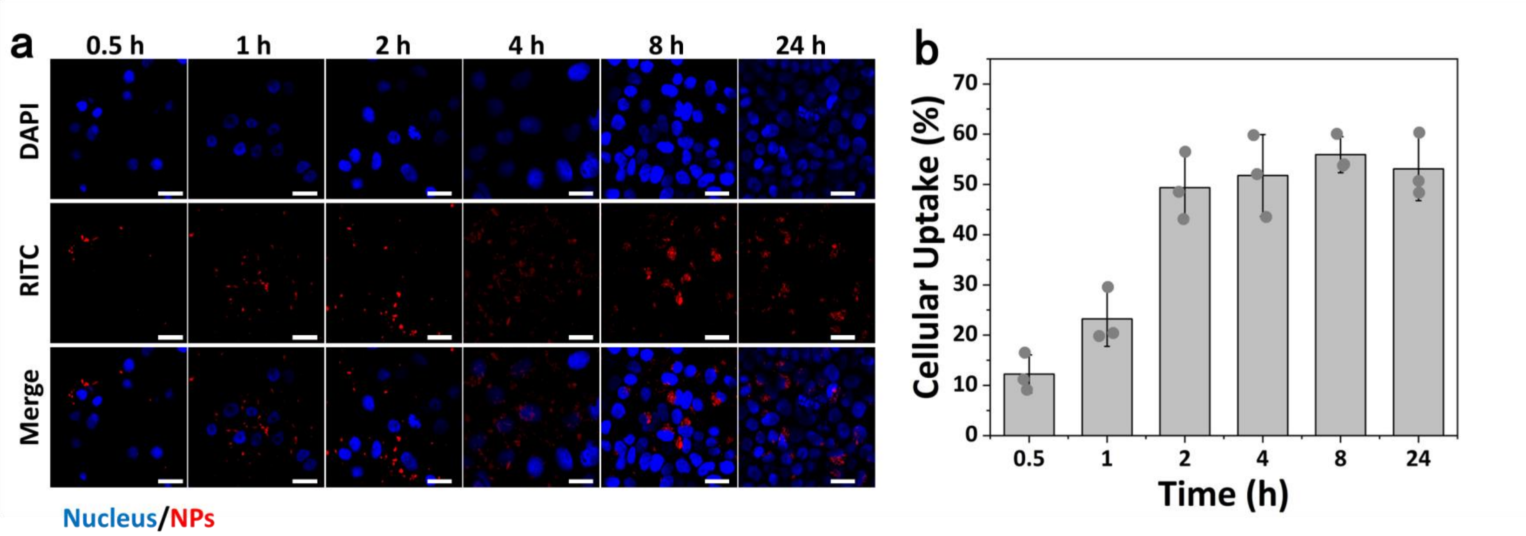

Supplementary Figure 27. Time-dependent cellular uptake of VSN. a CLSM images of the cellular uptake of RITC labeled VSN on Caco-2 cells at different times; blue: cell nucleus stained with DAPI, red: RITC labeled NPs, scale bar 20 μm. Experiment was repeated three times independently with similar results. **b** Cellular uptake amount (%) of VSN on Caco-2 cells at different times. Data are presented as the mean ± SD (n = 3 independent experiments). Source data are provided as a Source Data file.

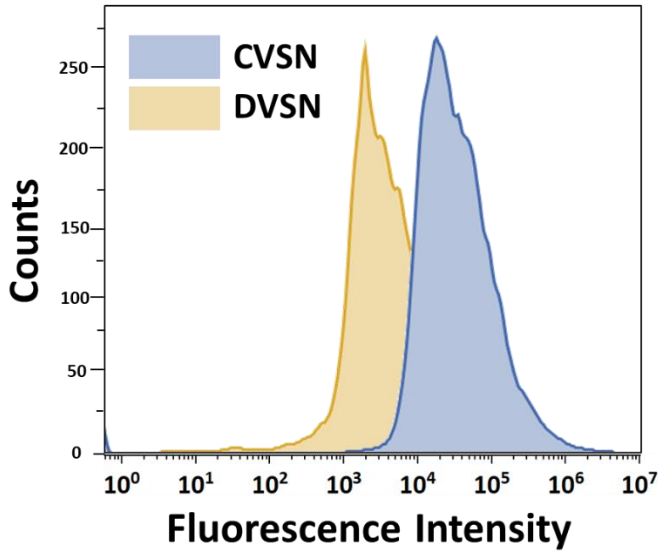

Supplementary Figure 28 Cellular uptake efficiency of NPs with different chirality. Cellular internalization of CVSN and DVSN on Caco-2 cells measured via FCM.

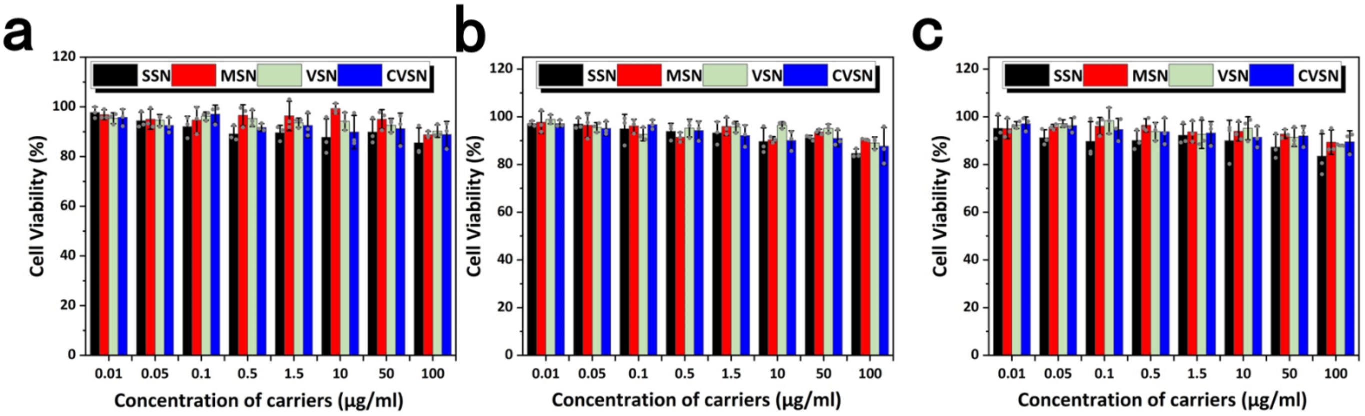

Supplementary Figure 29. Cytotoxicity of NPs. Cytotoxicity of NPs after incubation with Caco-2 cells for **a** 24, **b** 48 and **c** 72 h measured by Cell Counting Kit-8 (CCK-8) analysis. Data are presented as the mean ± SD (n = 3 independent experiments). Source data are provided as a Source Data file.

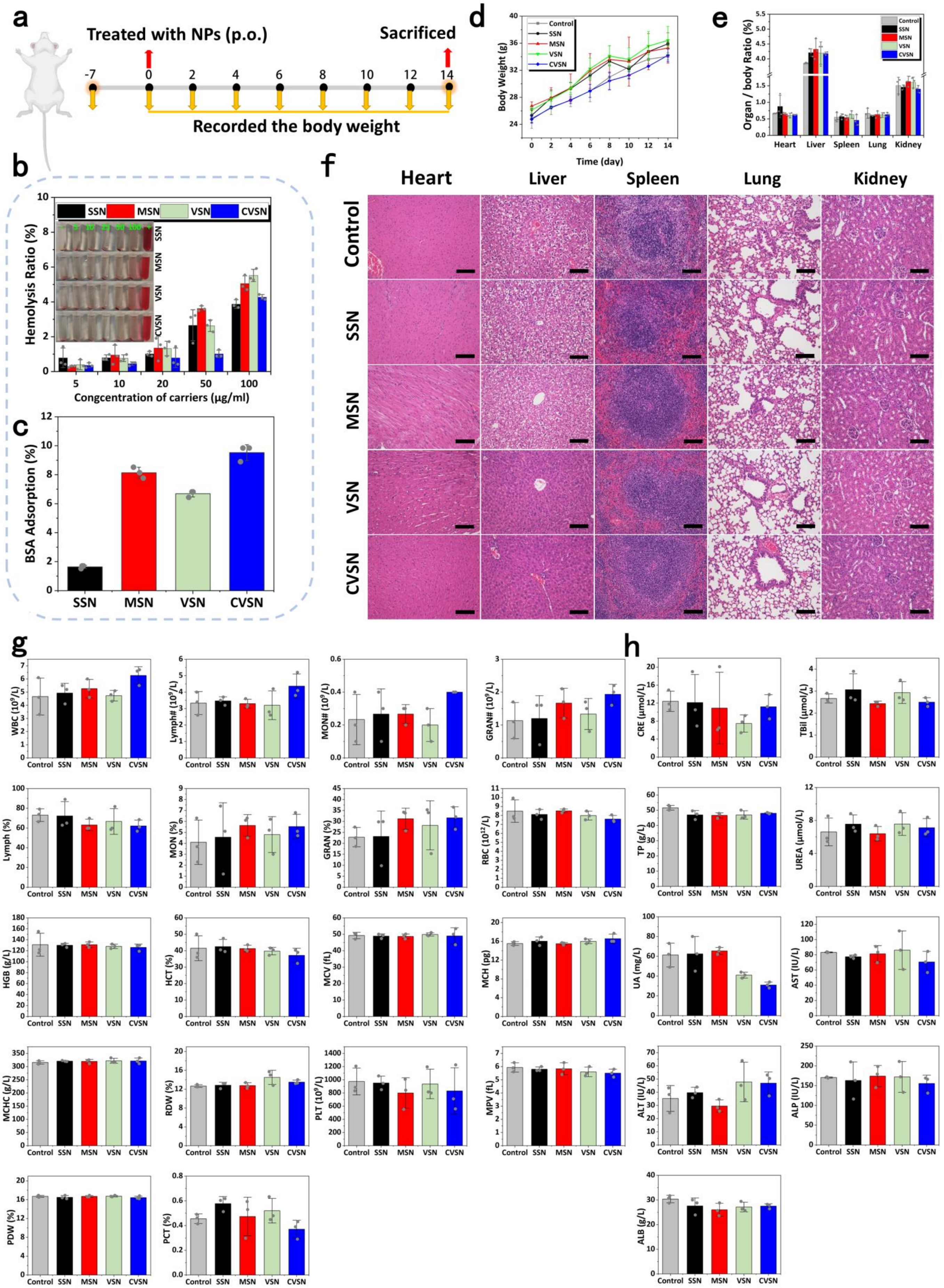

a
Treated with NPs (p.o.)
Sacrificed
-7
0
2
4
6
8
10
12
14
Recorded the body weight
b
Hemolysis Ratio (%)
SSN
MSN
VSN
CVSN
Congcentration of carriers (μg/ml)
c
BSA Adsorption (%)
SSN
MSN
VSN
CVSN
d
Body Weight (g)
Time (day)
Control
SSN
MSN
VSN
CVSN
e
Organ / body Ratio (%)
Heart
Liver
Spleen
Lung
Kidney
f
Heart
Liver
Spleen
Lung
Kidney
Control
SSN
MSN
VSN
CVSN
g
WBC (10^9/L)
Lymph# (10^9/L)
MON# (10^9/L)
GRAN# (10^9/L)
Lymph (%)
MON (%)
GRAN (%)
RBC (10^12/L)
HGB (g/L)
HCT (%)
MCV (fL)
MCH (pg)
MCHC (g/L)
RDW (%)
PLT (10^9/L)
MPV (fL)
PDW (%)
PCT (%)
h
CRE (μmol/L)
TBil (μmol/L)
TP (g/L)
UREA (μmol/L)
UA (mg/L)
AST (IU/L)
ALT (IU/L)
ALP (IU/L)
ALB (g/L)
Control
SSN
MSN
VSN
CVSN

Supplementary Figure 30. Biocompatibility of NPs. a Treatment protocols on the in vivo toxicity assessments of NPs.**b** Hemolytic photographs**,** hemolysis ratios and **c** BSA adsorption of NPs. Data are presented as the mean ± SD (n = 3 independent experiments). Source data are provided as a Source Data file. **d** The dynamic body weight, **e** organ/body ratios, **f** representative histopathological images (scale bar: 100 μm) of the major organs (Experiment was repeated three times independently with similar results.), **g** hematology results and **h** biochemical result of mice after oral treatment of NPs. Data are presented as the mean ± SD (n = 3 independent experiments). Source data are provided as a Source Data file.

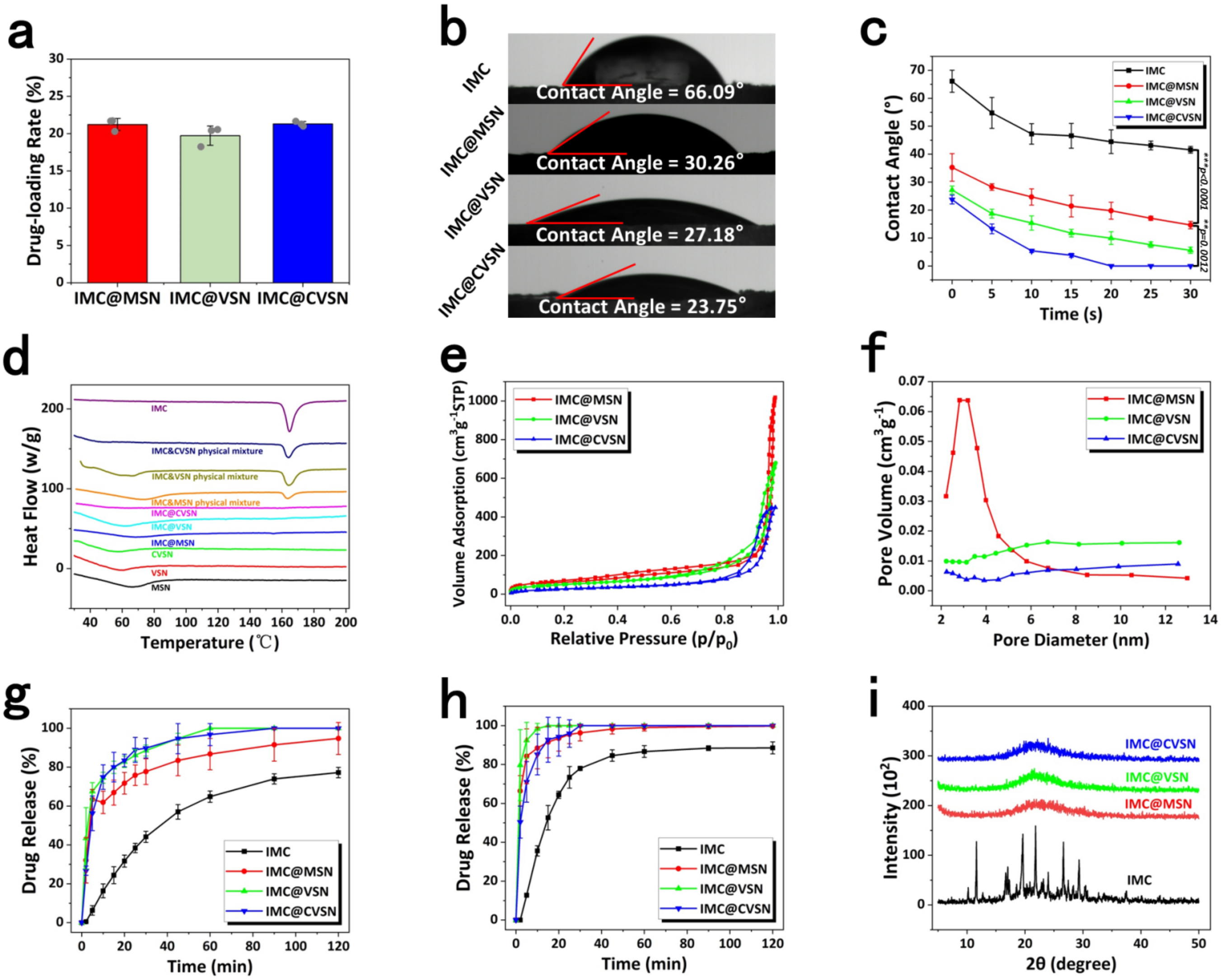

Supplementary Figure 31. Drug loading and release properties of NPs. a Drug loading capacity of IMC@MSN, IMC@VSN and IMC@CVSN. Data are presented as the mean ± SD (n = 3 independent experiments). Source data are provided as a Source Data file. **b** The initial contact angles and **c** contact angles-time curves of IMC@MSN, IMC@VSN and IMC@CVSN. Data are presented as the mean ± SD (n = 3

independent experiments). Source data are provided as a Source Data file. **d** Differential scanning calorimetry (DSC) curves of IMC@MSN, IMC@VSN and IMC@CVSN before and after drug loaded. Source data are provided as a Source Data file. **e** N_2 adsorption/desorption isotherms and **f** pore size distribution curves of IMC@MSN, IMC@VSN and IMC@CVSN. Source data are provided as a Source Data file. In vitro drug release of IMC@MSN, IMC@VSN and IMC@CVSN at **g** pH 6.5 PBS and **h** pH 7.4 PBS. Data are presented as the mean ± SD (n = 3 independent experiments). Source data are provided as a Source Data file. **i** XRD patterns of IMC loaded NPs after store at ambient temperature and pressure for 30 days. Source data are provided as a Source Data file.

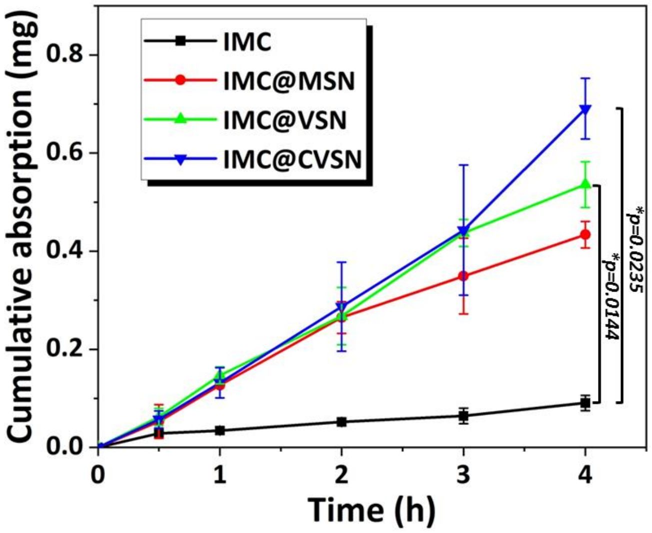

Supplementary Figure 32. Ex vivo intestinal transport of IMC@NPs. Cumulative transport amount of IMC via everted intestinal sacs model. Data are presented as the mean ± SD (n = 3 independent experiments). *P < 0.05 by two-tailed Student's t-test. Source data are provided as a Source Data file.

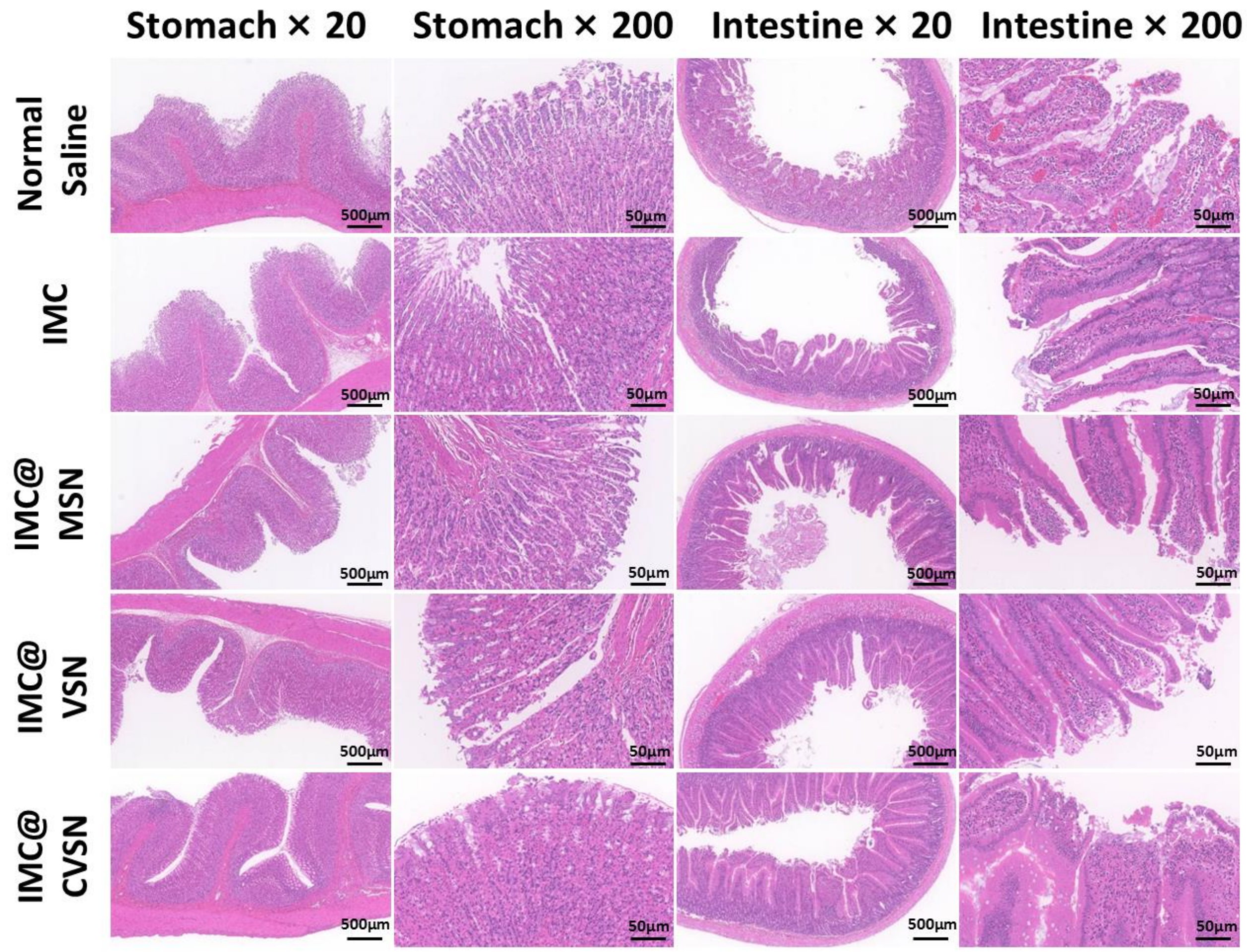

Supplementary Figure 33. GI tract irritation of IMC@NPs. Histopathological images of the stomach and intestinal tissues after oral treated with IMC loaded NPs. Experiment was repeated three times independently with similar results.

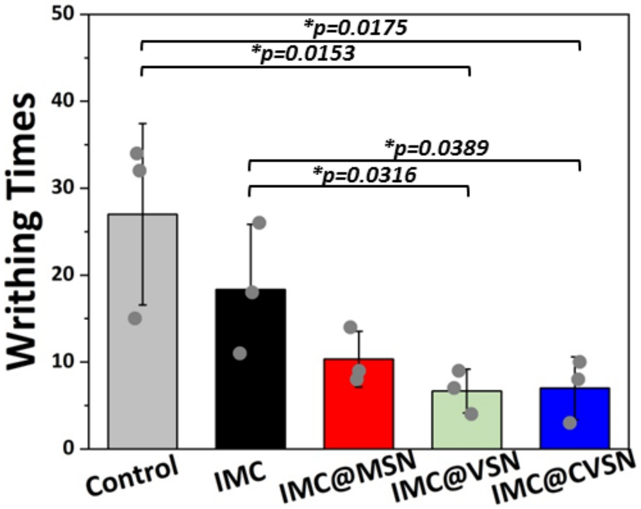

Supplementary Figure 34. **The analgesic effect of IMC@NPs.** The writhing times of IMC, IMC@MSN, IMC@VSN and IMC@CVSN on mouse writhing test (MWT). Data are presented as the mean ± SD (n = 3 independent experiments). *P < 0.05 by two-tailed Student’s t-test. Source data are provided as a Source Data file.

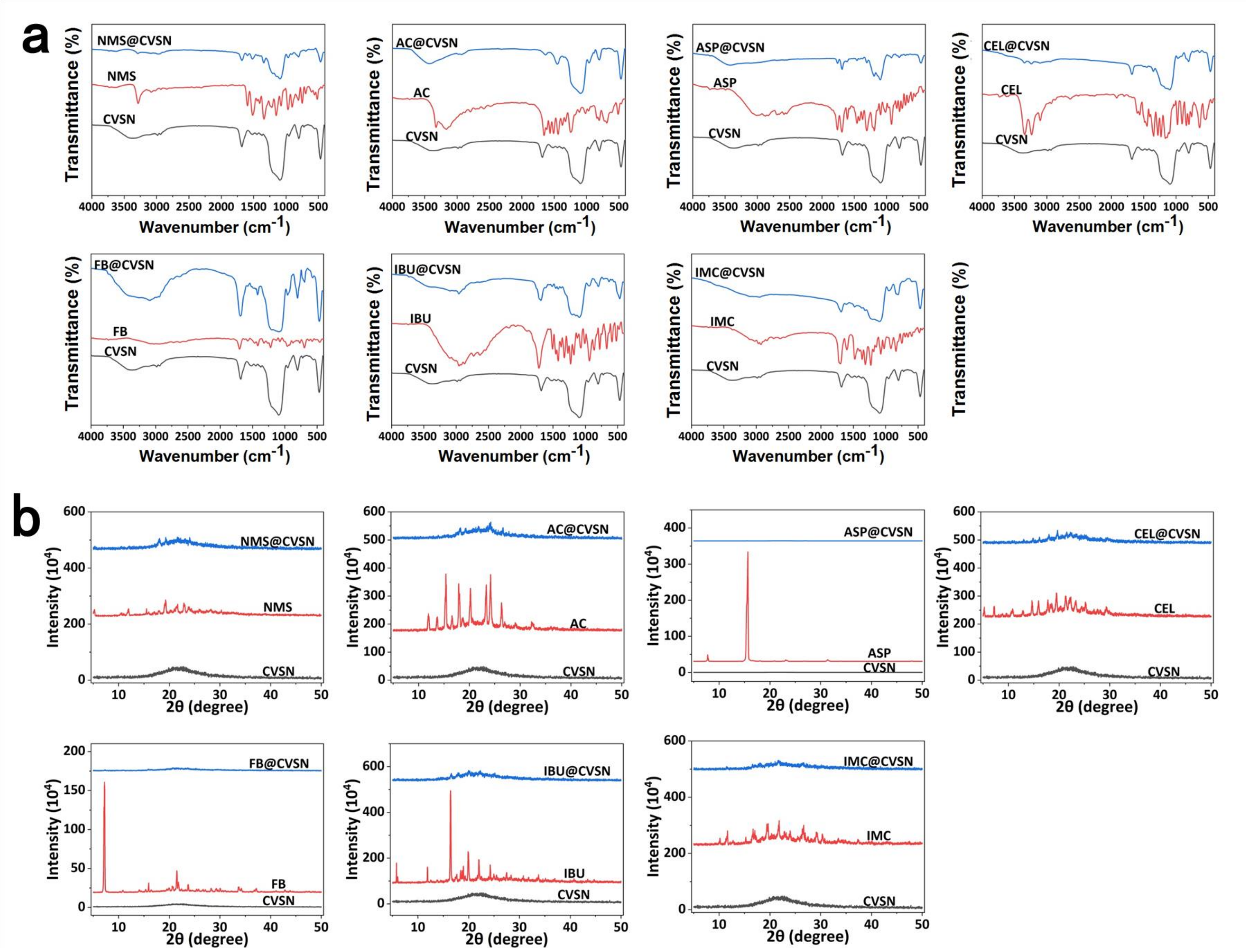

Supplementary Figure 35. Interactions between CVSN and NASIDs. **a** FTIR spectra and **b** XRD patterns of NMS@CVSN, AC@CVSN, ASP@CVSN, CEL@CVSN, FB@CVSN, IBU@CVSN and IMC@CVSN before and after drug loaded.

Supplementary Tables

Supplementary Table 1. Texture properties of IMC loaded NPs

Sample	S_{BET} (m^2/g)	V_t (cm^3/g)	W_{BJH} (nm)	C_d (%)
IMC@MSN	264.15	1.57	2.8	21.23±0.81
IMC@VSN	184.23	1.05	2.6	19.55±1.03
IMC@CVSN	101.30	0.69	2.2	21.30±0.32

Supplementary Table 2. Pharmacokinetic parameters obtained after oral administration of IMC and IMC loaded NPs

Pharmacokinetic parameters	IMC	IMC@MSN	IMC@VSN	IMC@CVSN
C_{max} (μg/ml)	24.9 ± 1.3	97.1 ± 3.3	179.9 ± 29.5	178.7 ± 14.1
T_{max} (h)	12	6	4	8
$T_{1/2}$ (h)	5.2 ± 1.3	14.5 ± 1.2	12.5 ± 1.7	10.7 ± 1.5
AUC_{0-t} (mg/L*h)	327.5 ± 45.1	1993.1 ± 77.9	3593.2 ± 465.5	3753.5 ± 290.8
MRT_{0-t} (mg/L*h)	12.20 ± 0.5	14.91 ± 0.4	15.9 ± 0.7	17.1 ± 0.4
F (%)	100.0	608.4	1096.9	1145.9

Data are presented as mean ± SD (n = 3)

Supplementary Table 3. Inhibition rate (%) of IMC loaded NPs in MAST

Inhibition rate (%)	MAST						
Sample	0.5 h	1 h	1.5 h	2 h	3 h	4 h	6 h
IMC@MSN	286.43 ±117.67	873.49 ±399.56	1315.91 ±325.85	1538.48 ±301.03	1602.76 ±310.59	1817.42 ±275.24	2033.68 ±130.18
IMC@VSN	214.70 ±184.78	1130.48 ±221.33	1607.96 ±126.43	1709.66 ±90.75	1856.41 ±106.46	2112.67 ±71.80	2297.58 ±29.31
IMC@CVSN	241.01 ±226.63	881.21 ±231.75	1428.90 ±45.79	1639.07 ±72.60	1822.11 ±165.04	2068.64 ±215.44	2315.86 ±26.71

Data are presented as mean ± SD (n = 3)

Supplementary Table 4. Inhibition rate (%) of IMC loaded NPs in MEST and MWT

Sample	MEST	MWT
IMC@MSN	133.33±10.29	192.31±37.09
IMC@VSN	156.42±21.00	234.62±29.04
IMC@CVSN	153.73±25.71	230.77±41.60

Data are presented as mean ± SD (n = 3)

Supplementary Table 5. Parameters of the NASIDs

Drug	Abbreviation	Structure	Classification	BCS
Nimesulide	NMS	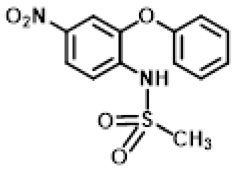	Other	2
Acetaminophen	AC	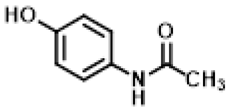	P-aminophenol	4
Aspirin	ASP	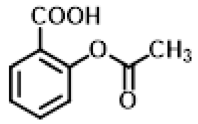	Salicylic acid	3
Celecoxib	CEL	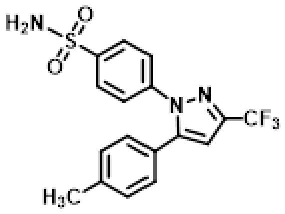	-coxib	2
Flurbiprofen	FB	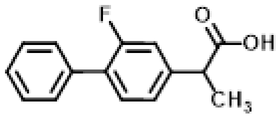	Propionic acid derivative	2
Ibuprofen	IBU	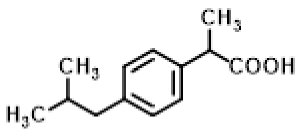	Propionic acid derivative	2
Indomethacin	IMC	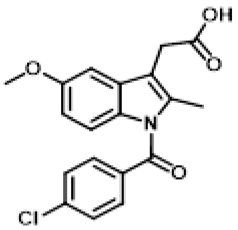	Acetic acid derivative	2

Supplementary Table 6. Inhibition rate (%) of NASIDs loaded CVSN in MEST.

Sample	MEST
NMS@CVSN	141.00 ±7.95
AC@CVSN	208.28 ±4.33
ASP@CVSN	121.12 ±16.48
CEL@CVSN	125.45 ±18.51
FB@CVSN	148.88 ±170.31
IBU@CVSN	142.89 ±10.61
IMC@CVSN	229.95 ±18.68

Data are presented as mean ± SD (n = 3)

Supplementary References

1. Chen X, et al. Chiral Nanosilica Drug Delivery Systems Stereoselectively Interacted with the Intestinal Mucosa to Improve the Oral Adsorption of Insoluble Drugs. ACS nano **17**, 3705-3722 (2023).
