# Supplementary material for: Nanoparticles exhibiting virus-mimic surface topology for enhanced oral delivery
Source: Nat Commun. 2023 Nov 24;14:7694. doi: 10.1038/s41467-023-43465-y (PMC10673925; doi:10.1038/s41467-023-43465-y)
Supplement: Supplementary file 3 — Reporting Summary [file 41467_2023_43465_MOESM3_ESM.pdf]

nature portfolio

Corresponding author(s): Heran Li

Last updated by author(s): Nov 1, 2023

Reporting Summary

Nature Portfolio wishes to improve the reproducibility of the work that we publish. This form provides structure for consistency and transparency in reporting. For further information on Nature Portfolio policies, see our Editorial Policies and the Editorial Policy Checklist.

Statistics

For all statistical analyses, confirm that the following items are present in the figure legend, table legend, main text, or Methods section.

n/a	Confirmed	
☐	☒	The exact sample size (*n*) for each experimental group/condition, given as a discrete number and unit of measurement
☐	☒	A statement on whether measurements were taken from distinct samples or whether the same sample was measured repeatedly
☐	☒	The statistical test(s) used AND whether they are one- or two-sided<br>*Only common tests should be described solely by name; describe more complex techniques in the Methods section.*
☒	☐	A description of all covariates tested
☒	☐	A description of any assumptions or corrections, such as tests of normality and adjustment for multiple comparisons
☒	☐	A full description of the statistical parameters including central tendency (e.g. means) or other basic estimates (e.g. regression coefficient) AND variation (e.g. standard deviation) or associated estimates of uncertainty (e.g. confidence intervals)
☐	☒	For null hypothesis testing, the test statistic (e.g. *F*, *t*, *r*) with confidence intervals, effect sizes, degrees of freedom and *P* value noted<br>*Give P values as exact values whenever suitable.*
☒	☐	For Bayesian analysis, information on the choice of priors and Markov chain Monte Carlo settings
☒	☐	For hierarchical and complex designs, identification of the appropriate level for tests and full reporting of outcomes
☒	☐	Estimates of effect sizes (e.g. Cohen's *d*, Pearson's *r*), indicating how they were calculated

Our web collection on statistics for biologists contains articles on many of the points above.

Software and code

Policy information about availability of computer code

Data collection	Zetasizer Software7.13; APPCIMain 8.60.160.11228; Brucker MI SE; Aperio ImageScope v12.3.3.3.5048; SlideViewer; ANASTAR5.2; ZEN 3.6(ZEN lite) v3.6.095.06000; BD Accuri C6 Plus Software1.0.23.1
Data analysis	Origin 2021 9.8.0.200; Image J 1.53e; DAS v2.0; Microsoft Office 2016; FlowJo_v10.8.1

For manuscripts utilizing custom algorithms or software that are central to the research but not yet described in published literature, software must be made available to editors and reviewers. We strongly encourage code deposition in a community repository (e.g. GitHub). See the Nature Portfolio guidelines for submitting code & software for further information.

Data

Policy information about availability of data

All manuscripts must include a data availability statement. This statement should provide the following information, where applicable:

- Accession codes, unique identifiers, or web links for publicly available datasets
- A description of any restrictions on data availability
- For clinical datasets or third party data, please ensure that the statement adheres to our policy

Source data are provided with this paper. Source data are available for Figs. 1b, f, 2, 3 b, d, e, g, k–l, 4c, d, f, 5c–f , j–n, 6c, d, f, 7, 8d–o, Supplementary Figs. 2–8, 10–21, 23, 24, 26–32, 34, 35 in the associated “Source Data” file. The source data have been deposited in the Figshare database (10.6084/m9.figshare.24476458). All the other data that support the findings of this study are available within the Article and its Supplementary Information files and from the corresponding author.

10.6084/m9.figshare.24476458

Research involving human participants, their data, or biological material

Policy information about studies with human participants or human data. See also policy information about sex, gender (identity/presentation), and sexual orientation and race, ethnicity and racism.

Reporting on sex and gender	This study did not involve clinic trial or disease research, sex and gender were identified as insignificant factor in results analysis. This study involves samples from 12 male and 8 female (60% male and 40% female).
Reporting on race, ethnicity, or other socially relevant groupings	We collected samples from Chinese patients for the ex vivo experiment for the adsorption of nanoparticles with no consideration on the race, ethnicity, or other socially relevant groupings of patient.
Population characteristics	The samples of ex vivo para-carcinoma tissues after clinical treatment were obtained from Chinese small intestinal stromal tumor (SIST) patients (male or female [determined the sex and/or gender of participants based on the record in medical records], aged larger than 18)
Recruitment	Paraneoplastic tissues were obtained from 20 Chinese small intestinal stromal tumor (SIST) patients (male or female, aged larger than 18) at the First Hospital of China Medical University, who informed consented to donate their biological samples and information.
Ethics oversight	The collection of patient samples was approved by the Ethics Committee of the China Medical University (No [2022]463, No 2023[64]).

Note that full information on the approval of the study protocol must also be provided in the manuscript.

Field-specific reporting

Please select the one below that is the best fit for your research. If you are not sure, read the appropriate sections before making your selection.

[x] Life sciences   [ ] Behavioural & social sciences   [ ] Ecological, evolutionary & environmental sciences

For a reference copy of the document with all sections, see nature.com/documents/nr-reporting-summary-flat.pdf

Life sciences study design

All studies must disclose on these points even when the disclosure is negative.

Sample size	The sample size was 3 for in vitro experiments, 3 for ex vivo experiments, and 3 for in vivo experiments. Sample size was chosen to ensure reproducibility of the experiments and to support meaningful conclusions. The sample size (n) of each experiment is provided in Figure legends in the Article and its Supplementary Information.
Data exclusions	No data were excluded from the analyses.
Replication	We confirmed that all repeated attempts were successful. Experiment repeat numbers are reported in Figure Legends.
Randomization	Samples were randomly allocated into experimental groups.
Blinding	The investigators were blinded to group allocation during data collection and analysis.

Reporting for specific materials, systems and methods

We require information from authors about some types of materials, experimental systems and methods used in many studies. Here, indicate whether each material, system or method listed is relevant to your study. If you are not sure if a list item applies to your research, read the appropriate section before selecting a response.

Materials & experimental systems

n/a	Involved in the study	
[x]	[ ]	Antibodies
[ ]	[x]	Eukaryotic cell lines
[x]	[ ]	Palaeontology and archaeology
[ ]	[x]	Animals and other organisms
[x]	[ ]	Clinical data
[x]	[ ]	Dual use research of concern
[x]	[ ]	Plants

Methods

n/a	Involved in the study	
[x]	[ ]	ChIP-seq
[ ]	[x]	Flow cytometry
[x]	[ ]	MRI-based neuroimaging

Eukaryotic cell lines

Policy information about cell lines and Sex and Gender in Research

Cell line source(s)	Caco-2 cells were provided by the Procell Life Science Technology Co. Ltd (CL-0005) and cultured in Dulbecco's modified Eagle medium (DMEM) containing 10% fetal bovine serum (Hy Clone, USA).
Authentication	The cell lines were certificated by the manufactures using short tandem repeat (STR) method.
Mycoplasma contamination	All cell lines tested negative for mycoplasma contamination by the manufactures using TransDetect PCR Mycoplasma Kit.
Commonly misidentified lines (See ICLAC register)	No commonly misidentified cell lines were used in the study.

Animals and other research organisms

Policy information about studies involving animals; ARRIVE guidelines recommended for reporting animal research, and Sex and Gender in Research

Laboratory animals	Institute of Cancer Research (ICR) mice (male, 6 weeks old, ~30 g), and Sprague-Dawley (SD) rats (male, 6 weeks old, ~200 g) were involved in the study, and received humane care. Animals were housed in equipped animal facility at a constant temperature (~25°C) under a dark/light cycle, treated with a standard diet and water, and fed adaptively for 1 week after arrival.
Wild animals	The study did not involve wild animals.
Reporting on sex	Sex has no been reported as a critical factor for oral adsorption of nanoparticles. Male mice were used in this study.
Field-collected samples	The study did not involve samples collected from the field.
Ethics oversight	All animal experiments were approved by the Animal Ethics Committee of China Medical University (Protocol Number: CMUKT2022255), and were performed in accordance with the guidelines for the Care and Use of Laboratory Animals.

Note that full information on the approval of the study protocol must also be provided in the manuscript.

Flow Cytometry

Plots

Confirm that:

- [x] The axis labels state the marker and fluorochrome used (e.g. CD4-FITC).
- [x] The axis scales are clearly visible. Include numbers along axes only for bottom left plot of group (a 'group' is an analysis of identical markers).
- [x] All plots are contour plots with outliers or pseudocolor plots.
- [x] A numerical value for number of cells or percentage (with statistics) is provided.

Methodology

Sample preparation	Caco-2 cells were provided by the Procell Life Science Technology Co. Ltd (CL-0005)
Instrument	Flow Cytometer（BD Accuri C6 Plus）
Software	Flowjo_v10 and BD Accuri C6 Plus Software
Cell population abundance	Only Caco-2 cells was used for analysis.
Gating strategy	Elliptic gates on FSC-A and SSC-A signals were used to exclude cell debris and clumps.

- [x] Tick this box to confirm that a figure exemplifying the gating strategy is provided in the Supplementary Information.
